# AI Empathy as a Human Disposition in Generative AI Interaction: Development and Preliminary Psychometric Evaluation of a Cognitive–Affective–Motivational Scale

**DOI:** 10.3390/bs16101717

**Published:** 2026-09-22

**Authors:** Sukjae Joshua Kang, Juyoung Lee, Seong-Joo Kang

**Affiliations:** 1 Peptide Biology Laboratory, The Salk Institute for Biological Studies, La Jolla, CA 92037, USA; sjkang@salk.edu; 2 Department of Gifted Education, Seoul National University of Education, Seoul 06639, Republic of Korea; leejyssam@gmail.com; 3 Department of Science Education, Korea National University of Education, Cheongju-si 28173, Republic of Korea

**Keywords:** AI empathy, generative artificial intelligence, human–AI interaction, cognitive empathy, affective empathy, motivational empathy, individual differences, scale development

## Abstract

Generative artificial intelligence (GenAI) is increasingly embedded in human reasoning and problem solving, yet individuals differ in how they interpret AI behavior, respond affectively to its limitations, and regulate subsequent interaction. This study developed and preliminarily evaluated the 42-item AI Empathy Scale for Generative AI Interaction (AIES-G) as a measure of these human dispositions. The provisional 3 × 2 framework crosses cognitive, affective, and motivational domains with other- and self-oriented responses, yielding six subfactors: Perspective Taking, Imaginative Self-Projection, Empathic Concern, Personal Distress, Prosocial Intention, and Reflective Inquiry. Participants were 201 individuals from four educational cohorts who completed the AIES-G before and after GenAI-supported activities. Exploratory factor analysis of the 42 post-test items indicated adequate factorability (KMO = 0.859; Bartlett’s χ^2^(861) = 4225.91, *p* < 0.001). Parallel analysis and Velicer’s MAP criterion supported six factors (48.2% of item variance), but one factor mainly reflected negatively keyed items, and Prosocial Intention formed no distinct factor. Internal consistency was acceptable to strong across the six subscales and total scale. Scores excluding Personal Distress increased from pre-test to post-test (dz = 1.46), possibly reflecting partial overlap with instructed activities. Increases were larger for Perspective Taking, Imaginative Self-Projection, Prosocial Intention, and Reflective Inquiry than for Empathic Concern, whereas Personal Distress met the prespecified equivalence criterion. Findings provide preliminary psychometric evidence for a proposed multidimensional human disposition relevant to behavioral research on human–AI interaction; confirmatory, behavioral, and cross-context validation remains necessary.

## 1. Introduction

### 1.1. Generative AI as a Behavioral Context

Generative artificial intelligence (GenAI) has become an increasingly important context for human reasoning, problem solving, communication, learning, and decision making. Because GenAI produces probabilistic and context-sensitive responses to user prompts, interaction is typically iterative: users formulate requests, interpret outputs, identify discrepancies, and decide whether to accept, revise, verify, or reject AI-generated information (Chiu et al., 2023; Kasneci et al., 2023). These activities require more than technical proficiency; they also involve cognitive interpretation, affective response, and decisions about whether and how to continue the interaction.

A prototypical example is a user who receives a fluent but questionable GenAI response and then tries to understand how the prompt or context may have shaped it, considers how the output could affect another person, regulates frustration, and decides whether to verify, revise, or continue the interaction. In this study, such a pattern illustrates AI empathy as a human orientation within an AI-mediated situation, not empathy toward AI as a sentient target.

These interactional demands are intensified by well-documented limitations of contemporary GenAI. Large language models can generate fluent but unsupported or factually incorrect outputs, including confabulations that are difficult for users to detect (Farquhar et al., 2024). GenAI systems can also reproduce or amplify biases originating in training data, model development, and deployment contexts, with consequences for fairness and representation (Afreen et al., 2025). At the same time, the internal processes that produce a particular generative output are often difficult for end users to inspect directly, which has motivated recent work on explainable generative AI and on explanation criteria such as verifiability and interactivity (Schneider, 2024). Hallucination, bias, and limited transparency therefore make users’ interpretation, affective response, and subsequent regulation of AI-mediated interaction behaviorally consequential.

Individuals with comparable AI knowledge may nevertheless respond differently to the same inaccurate, ambiguous, biased, or poorly contextualized output. Some users attempt to reconstruct how the response was generated, some experience frustration or uncertainty, and others focus on possible consequences for themselves or other people and decide whether to revise or verify the output. Such variation motivates a behavioral question that is not reducible to AI literacy alone: how do users differ in the ways they interpret, emotionally respond to, and regulate engagement in AI-mediated situations?

### 1.2. Research Problem and Construct Focus

The present study addresses this question through the construct of AI empathy. AI empathy is defined here as a multidimensional human disposition expressed within GenAI-mediated interaction. The construct does not imply that GenAI possesses consciousness, emotion, intention, suffering, or moral patienthood. GenAI is therefore not treated as the literal recipient of empathy; rather, it is the interactional context that elicits a coordinated pattern of human cognitive interpretation, affective appraisal, and motivational regulation. Within that context, the locus of attention differs by subfactor: users may functionally model system behavior, respond to consequences for other people, or monitor and regulate their own reactions and inquiry. The common mechanism proposed across the six dimensions is this coordinated orientation to AI-mediated uncertainty, discrepancy, and consequence, expressed through cognitive, affective, and motivational processes. Accordingly, the central issue is not whether GenAI itself is empathic, but whether human users show systematic individual differences in these orientations. The label AI empathy is retained because the framework is explicitly derived from multidimensional empathy theory and preserves perspective-related, affective, self–other, and action-readiness components while functionally reconstructing them for a nonsentient AI-mediated context; it should not be interpreted as empathy directed toward AI itself.

The specific construct gap addressed here is the lack of a single multidimensional measure that organizes users’ cognitive interpretation, affective responses, and self- and other-oriented motivational regulation within GenAI-mediated interaction.

The detailed theoretical basis for this construct—including its relationship to multidimensional empathy, the self–other distinction, motivated engagement, and adjacent constructs—is developed in the Literature Review (Section 2). The Introduction therefore limits itself to the research problem and the study purpose rather than reproducing the full conceptual framework or scale-development rationale.

### 1.3. Purpose of the Study

The purpose of the present study was to develop and preliminarily evaluate the 42-item AI Empathy Scale for Generative AI Interaction (AIES-G) as a measure of individual differences in cognitive, affective, and motivational dispositions during GenAI-mediated interaction.

To avoid confusion with the previously published Authentic and Inauthentic Expression Scale, which also uses the acronym AIES (Al-Khouja et al., 2022), the present instrument is referred to hereafter as the AI Empathy Scale for Generative AI Interaction (AIES-G).

## 2. Literature Review

### 2.1. Multidimensional Foundations of Empathy

Empathy has long been conceptualized as a multidimensional rather than unitary construct. A major foundation for this view is Davis’s (1983) Interpersonal Reactivity Index (IRI), which distinguished Perspective Taking, Fantasy, Empathic Concern, and Personal Distress. The enduring contribution of the IRI lies not simply in identifying four subscales but in demonstrating that cognitive understanding, imaginative projection, other-oriented concern, and self-oriented discomfort represent functionally different processes.

Perspective Taking refers to the tendency to adopt or reconstruct another standpoint. Fantasy represents imaginative self-projection into represented situations. Empathic Concern refers to other-oriented feelings of warmth, compassion, and concern, whereas Personal Distress reflects self-oriented discomfort in emotionally difficult circumstances. The distinction between Empathic Concern and Personal Distress is particularly important because affective activation does not necessarily lead to constructive engagement. Consistent with the distinction between empathic concern and self-oriented personal distress, other-oriented concern may facilitate approach and helping, whereas intense self-focused distress may contribute to avoidance or withdrawal (Batson et al., 1987; Davis, 1983).

Subsequent instruments have further refined the cognitive–affective architecture of empathy. The Questionnaire of Cognitive and Affective Empathy distinguishes broad cognitive and affective dimensions while differentiating more specific processes within each domain (Reniers et al., 2011). The Empathy Components Questionnaire distinguishes empathic ability from empathic drive, thereby recognizing that possessing the capacity to understand or respond is not equivalent to being motivated to exercise that capacity (Batchelder et al., 2017).

These developments support several premises underlying the AIES-G. First, empathy includes partially separable cognitive, affective, motivational, and regulatory processes. Second, the direction of attention matters: some responses extend beyond the individual toward an interactional counterpart, a shared activity, or the welfare of other people, whereas others remain centered on the individual’s own simulation, discomfort, regulation, or learning. Third, understanding and emotional responsiveness do not necessarily culminate in constructive action.

These frameworks establish that empathy is neither unitary nor reducible to affective resonance alone, but they were developed primarily for human–human contexts. Applying their functional distinctions to GenAI-mediated learning therefore requires theoretical reconstruction rather than simple transfer.

### 2.2. Reconstructing Empathy for Human–GenAI Interaction

Research on AI and empathy has addressed several distinct phenomena, including whether users perceive artificial agents as empathic, whether people experience empathy toward robots or AI-authored narratives, and how AI can scaffold empathic communication between humans (Hancock et al., 2020; Hohenstein et al., 2023; Riek et al., 2009; Sharma et al., 2023; Shen et al., 2024). The present study focuses on a different target: the human user’s orientation within GenAI-mediated educational interaction. In this framework, references to the “perspective” of AI are functional rather than ontological. They refer to users’ attempts to model how a system appears to interpret prompts, constraints, context, examples, and task demands, not to any claim that the system has subjective experience, emotion, or a human-like mind. This distinction separates AI empathy from anthropomorphism while preserving the importance of human agency and responsibility in evaluating, revising, verifying, or rejecting AI-generated outputs. In this sense, the term AI empathy identifies the AI-mediated context rather than a single sentient target of empathy: PT and ISP concern functional reconstruction or simulation of the interaction, EC and PI concern consequences for other human users, and PD and RI concern the user’s own affective and reflective regulation. Recent evidence also cautions against transferring human–human empathy assumptions directly to human–AI interaction (HAI). People report different empathic responses to human- versus AI-authored experiences, and transparency about AI authorship can alter those responses (Shen et al., 2024). Conversely, AI-generated supportive messages can be rated as highly compassionate even though the system does not possess human emotional experience (Ovsyannikova et al., 2025). These findings indicate that perceived empathic quality, attribution of experience, and the user’s relation to the interaction partner are separable in HAI; the present framework therefore reconstructs functional components of empathy rather than treating a nonsentient GenAI system as interchangeable with a human social target.

Perspective Taking also intersects with human-centered explainable AI (XAI). Recent XAI reviews emphasize that useful explanations should support users’ understanding and mental models of system behavior, and increasingly frame explanation as interactive rather than as one-way disclosure (Rong et al., 2024; Raees et al., 2024). The AIES-G Perspective Taking construct is related to this goal but is not a measure of explanation quality: it assesses the user’s dispositional tendency to reconstruct how prompts, context, constraints, and available information may have contributed to a GenAI response.

The model crosses cognitive, affective, and motivational domains with other- and self-oriented responses, yielding six interrelated subfactors.

Guiding questions for the six subfactors are as follows: PT—What prompt, context, or constraint may have shaped this response? ISP—What information or reasoning would I need to construct an appropriate response? EC—How could this AI-mediated output affect another person? PD—How am I reacting emotionally to this problematic or uncertain output? PI—What could I do to improve the outcome for other people? RI—What should I reconsider or investigate further for myself? (Figure 1).

### 2.3. Motivational Empathy as a Parallel Domain of Engagement

Traditional multidimensional approaches have emphasized cognitive and affective components of empathy. However, cognitive understanding and affective responsiveness do not guarantee constructive action. A user may correctly identify why an AI-generated explanation is inappropriate without revising it. Another may feel concern about a biased or inaccurate response but fail to verify or improve it. A learner may also experience strong self-focused frustration that leads to avoidance rather than inquiry.

Motivated accounts of empathy address this gap by emphasizing that individuals regulate whether, when, toward whom, and at what cost they engage empathically. Empathic engagement is shaped by goals, values, perceived effort, anticipated rewards, and situational demands rather than operating as an invariant or entirely automatic response (Weisz & Zaki, 2018; Zaki, 2014). People may possess the capacity to understand another perspective and may even experience an affective response, yet still decide not to engage further.

The motivational dimension is especially salient in GenAI-mediated learning because interaction with a generative system is iterative. An initial output rarely represents the final educational event. It becomes an object for clarification, comparison, verification, revision, explanation, extension, or rejection. Productive interaction frequently depends on whether the user chooses to continue after encountering an incomplete, unexpected, or incorrect response.

The decision to continue constructively is therefore not peripheral to GenAI-mediated activity. Reformulating a prompt can require the user to clarify the task; verifying a claim can require comparison with external evidence; revising an explanation for another learner can require attention to the audience; and using an AI-generated discrepancy can prompt further inquiry. These examples illustrate a motivational function—readiness to initiate, sustain, or redirect constructive engagement—that may interact with cognitive and affective processes but is not defined as their downstream consequence.

The present framework therefore conceptualizes motivational empathy as a third, parallel higher-order domain. Motivational empathy refers to a distinct dispositional readiness to initiate, sustain, or redirect constructive engagement in AI-mediated situations. It is conceptually coordinated with cognitive interpretation and affective response but is not defined as a downstream product of either. It does not represent completed behavior or guarantee effective performance. Within the 3 × 2 framework, Prosocial Intention represents other-oriented action readiness aimed at improving outcomes for other people, whereas Reflective Inquiry represents self-oriented readiness to continue examining one’s own questions, assumptions, and judgments.

The inclusion of Prosocial Intention and Reflective Inquiry is not justified simply because they co-occur with cognitive and affective processes. Each has an independent motivational basis. Prosocial Intention draws on prosocial and empathic-motivation research that distinguishes readiness to benefit others from cognitive understanding or affective concern alone (Grant, 2008; Weisz & Zaki, 2018). Reflective Inquiry draws on self-regulated learning and metacognitive regulation, which emphasize sustained monitoring, reflection, and strategic continuation of inquiry (Panadero, 2017; Schraw & Dennison, 1994). Their shared function within the AIES-G is action readiness in AI-mediated situations, while their orientations differ: PI is directed toward improving outcomes for other people, and RI toward sustaining and deepening one’s own inquiry.

The distinction between disposition and behavior remains essential. Self-report and behavioral measures often correlate only modestly because they represent different response processes and levels of analysis (Dang et al., 2020). Meta-analytic findings similarly indicate that self-reported cognitive empathy should not be treated as a direct proxy for behavioral empathic ability (Murphy & Lilienfeld, 2019). The AIES-G is therefore designed to assess self-perceived orientation and readiness, not directly observed competence or actual AI-use behavior. In the present study, the term disposition denotes a relatively enduring but context-sensitive tendency rather than an immutable trait. Accordingly, a substantial short-term score change can indicate that self-perceived orientation or readiness has shifted within a particular learning context, but it does not by itself demonstrate durable trait change. The large Perspective Taking score change observed here (dz = 1.59) therefore raises a trait–state question that cannot be resolved with the present pre-test–post-test design. Temporal stability should be evaluated in future studies using test–retest intervals without targeted GenAI instruction and, ideally, longitudinal models that separate stable between-person differences from context-sensitive within-person change.

### 2.4. Self- and Other-Oriented Responses in AI-Mediated Activity

The proposed framework differentiates other-oriented and self-oriented responses across cognitive, affective, and motivational domains. These orientations are defined operationally rather than ontologically. The terms do not imply that a GenAI system is equivalent to a human interaction partner or should be treated as a moral patient possessing consciousness or suffering.

Other-oriented responses are defined by the locus of regulatory attention rather than by treating GenAI as a human-like recipient of empathy. They direct attention beyond the user’s immediate internal state toward an external aspect of the AI-mediated situation. In the cognitive domain, Perspective Taking reconstructs the system’s functional interpretation of prompts and constraints; in the affective domain, Empathic Concern is directed toward other people who may be affected by AI-generated outputs; and in the motivational domain, Prosocial Intention concerns readiness to improve AI-mediated outcomes for those people.

Thus, the ‘other’ pole is not a claim that all three subfactors share the same interpersonal target. Rather, it denotes an outward locus of attention: functional system behavior for PT and human consequences for EC and PI. This operational definition preserves the distinction between modeling a nonsentient system and empathic concern or prosocial motivation directed toward human beneficiaries.

Self-oriented responses are defined by a locus centered on the user’s own cognitive, affective, or motivational regulation. Imaginative Self-Projection involves mentally simulating the information or reasoning needed to construct an appropriate response; Personal Distress concerns the user’s own anxiety, frustration, confusion, or loss of control; and Reflective Inquiry concerns the user’s readiness to reconsider assumptions, reconstruct understanding, and sustain personal inquiry.

The distinction prevents self-orientation from being treated as uniformly maladaptive. Personal Distress may interfere with engagement when self-focused discomfort becomes dominant, but Reflective Inquiry may support learning by prompting users to reconsider assumptions and persist through uncertainty. Imaginative Self-Projection may similarly facilitate more effective prompting by helping users anticipate what information or constraints are absent.

Other-orientation is also not automatically beneficial. Concern about the consequences of an output may be based on inaccurate understanding, while a prosocial intention may not produce effective action. Constructive outcomes still require critical evaluation, relevant knowledge, and appropriate action.

The self–other axis should consequently be understood as an operational organizing principle describing the locus of attention and regulation, not as three pairs of identical targets or bipolar opposites. Other- and self-oriented responses may coexist and vary independently. Across the six subfactors, their commonality lies in how users orient and regulate cognition, affect, and motivation when AI-mediated uncertainty, discrepancy, or human consequence becomes salient.

### 2.5. A Three-by-Two Model of AI Empathy

Crossing the three domains of cognitive, affective, and motivational empathy with other- and self-oriented responses yields six theoretically defined subfactors. Perspective Taking refers to the tendency to consider how GenAI may interpret prompts, contextual information, and task constraints. It involves constructing a functional model of the system’s processing position.

Imaginative Self-Projection involves mentally simulating what information, specifications, or reasoning would be needed if one were attempting to generate an appropriate response from the system’s functional position. Whereas Perspective Taking is directed toward reconstructing the system’s apparent interpretation, Imaginative Self-Projection emphasizes the user’s own task-relevant simulation.

Empathic Concern refers to other-oriented sensitivity to the consequences of AI limitations, inaccuracies, or inappropriate outputs. It includes concern about how AI-generated materials may affect learners, peers, or other users.

Personal Distress refers to self-oriented anxiety, frustration, confusion, or perceived loss of control arising during problematic AI interaction. Unlike the other five subfactors, a high level of Personal Distress should not automatically be interpreted as a desirable outcome. Distress may alert a user to uncertainty or error, but excessive distress may inhibit constructive engagement.

Prosocial Intention concerns readiness to improve AI-mediated outcomes for others, encourage responsible use, or support other users through revision, explanation, or verification.

Reflective Inquiry refers to readiness to use interaction with GenAI as an opportunity to deepen personal inquiry, reconsider assumptions, and reconstruct understanding.

The model does not assume that the six subfactors are independent. Cognitive modeling, affective response, and motivated engagement may interact dynamically within the same episode of GenAI use. The relative salience of the six processes may vary across situations, individuals, and educational activities.

The six dimensions may also coexist with a broader AI-empathy disposition. Such a disposition would represent a general tendency to engage with GenAI in a cognitively attentive, affectively responsive, and constructively motivated manner. However, the existence and form of a higher-order disposition require empirical evaluation through comparisons among correlated-factor, higher-order, and bifactor models (Reise, 2012). The 3 × 2 model should therefore be understood as a theoretically structured but empirically testable representation.

### 2.6. Distinguishing AI Empathy from Adjacent Constructs

AI empathy is intended to complement rather than replace adjacent constructs such as AI literacy, anthropomorphism, AI attachment, trust in automation, self-regulated learning, emotion regulation, prosocial motivation, metacognition, epistemic cognition, and critical thinking in AI use. These constructs overlap with the AIES-G because GenAI-mediated educational activity requires users to evaluate AI capabilities, regulate uncertainty, monitor their own thinking, consider ethical consequences, and decide whether to continue, revise, verify, or reject AI-generated outputs. However, the distinctive focus of AI empathy is the coordinated orientation toward the system’s functional interpretation, the affective and human consequences of AI-mediated interaction, and constructive self- or other-oriented engagement. Epistemic cognition is particularly relevant to Reflective Inquiry because it concerns how individuals evaluate knowledge, evidence, certainty, and justification (Greene et al., 2016). However, the AIES-G extends beyond epistemic judgment by incorporating affective responses and self- and other-oriented motivational readiness within AI-mediated interaction. Recent measurement research has also introduced the Generative AI Engagement Scale (GAIES), which assesses the frequency of GenAI use and interaction styles such as Questioningness, Expressiveness, and Preciseness (Zhang et al., 2025). This construct is complementary to, but distinct from, AI empathy: GAIES focuses on how often and how users interact with GenAI, whereas the AIES-G focuses on cognitive interpretation, affective responsiveness, and self- and other-oriented motivational readiness during such interaction. AI attachment emphasizes emotional closeness, social substitution, and normative regard toward AI (Kasturiratna & Hartanto, 2026), whereas the AIES-G does not require relational attachment or moral regard toward AI. Critical thinking in AI use emphasizes verification, motivation to understand AI, and reflection on responsible AI use (Lau et al., 2026); the AIES-G overlaps with these processes but also includes affective and self- and other-oriented motivational responses. The Artificial Social Agent Questionnaire (ASAQ) provides a complementary standardized evaluation of users’ experiences with artificial social agents across multiple agent and interaction attributes (Fitrianie et al., 2025); in contrast, the AIES-G is intended to assess the user’s own cognitive, affective, and motivational disposition during GenAI-mediated interaction.

The proposed construct should therefore be understood as a configuration of related but theoretically distinguishable psychological dispositions rather than as a replacement for AI literacy, metacognition, epistemic cognition, prosocial motivation, or technology-related anxiety. AI literacy primarily concerns knowledge and competencies for understanding and using AI; metacognition concerns monitoring and regulation of one’s own cognition; epistemic cognition concerns judgments about knowledge, evidence, certainty, and justification; prosocial motivation concerns intentions to benefit others; and technology-related anxiety concerns apprehension or discomfort associated with technology use. The AIES-G instead examines how cognitive interpretation, affective response, and motivational readiness are jointly organized during interaction with a generative system. These conceptual distinctions are summarized in Table 1. Demonstrating empirical discriminability from these neighboring constructs remains an important objective for subsequent validation.

### 2.7. The Measurement Gap

Existing empathy instruments provide important theoretical foundations but were not designed for GenAI-mediated educational interaction. Measures such as the IRI, the Questionnaire of Cognitive and Affective Empathy, and the Empathy Components Questionnaire assess multidimensional empathy primarily in interpersonal settings (Batchelder et al., 2017; Davis, 1983; Reniers et al., 2011). They do not directly represent the iterative interpretation, uncertainty, error diagnosis, revision, and verification required when users interact with a generative system.

Technology-related empathy research has generally focused on different measurement targets. Some studies examine whether users perceive an AI system as empathic. Others investigate empathy toward artificial agents, AI-authored narratives, robots, or fictional characters. Research on digitally mediated empathy may also examine empathy expressed between human communicators through technological platforms (Collins et al., 2024; Shen et al., 2024). These targets differ from the human cognitive, affective, and motivational orientation assessed by the AIES-G.

Recent AI-specific measures already address some related processes; for example, critical thinking in AI use includes verification, motivation to understand AI, and reflection (Lau et al., 2026). The remaining measurement gap is particularly evident at the motivational level. GenAI users repeatedly decide whether to reformulate prompts, verify claims, revise misleading explanations, support other users, or sustain personal inquiry. Existing interpersonal empathy scales do not directly represent this action-oriented readiness in a GenAI-mediated context.

A second gap concerns the self–other distinction. GenAI interaction can elicit attention to the system’s functional processing, concern about consequences for others, self-focused distress, imaginative simulation, prosocial intention, and personal reflective inquiry. Existing scales do not organize these responses within a common domain-by-orientation framework.

The AIES-G addresses these gaps by reconstructing multidimensional empathy for interaction with a responsive but nonsentient system. It explicitly represents cognitive, affective, and motivational responses across other- and self-oriented forms of engagement. The instrument is intended to complement AI-literacy measures by assessing not only what users know and can do with AI, but also how they orient cognition, affect, responsibility, and motivation during AI-mediated educational activity.

### 2.8. The Present Study

The goal of the present study was to provide a preliminary psychometric evaluation of the proposed AIES-G framework by examining its exploratory structure, score reliability, and responsiveness across four educational cohorts (total N = 201). The study addressed the following research questions:

RQ1. To what extent do the exploratory structural and reliability results correspond to the proposed six-factor AIES-G framework?
RQ2. How responsive are total, domain, and subfactor AIES-G scores to sustained GenAI-mediated educational experiences?
RQ3. Is the direction of pre-test–post-test change in the total AIES-G score observed across the four educational cohorts and consecutive semesters?

These research questions concern preliminary psychometric structure and score responsiveness; they do not test causal intervention effects or directly observed empathic behavior. Participant, cohort, procedure, and administration details are reported in Section 3.

## 3. Materials and Methods

### 3.1. Research Design

This study employed a pre-test–post-test repeated-measures design embedded in four intact educational cohorts implemented across two consecutive academic semesters. The design served two related purposes. First, it examined the preliminary psychometric properties of AIES-G, including its exploratory factor structure and internal consistency. Second, it evaluated whether the instrument was responsive to changes occurring during course-embedded educational activities involving GenAI.

The psychometric analyses used the full sample of 201 participants with complete pre-test and post-test responses. The responsiveness analyses examined changes in the total AIES-G score, the three higher-order domain scores, and the six subfactor scores across the instructional period. Comparisons across educational contexts were conducted primarily among Groups 1–3 because these cohorts participated concurrently during the Fall 2025 semester. Group 4, which participated during the Spring 2026 semester, was treated as a subsequent-semester cohort used to examine whether the direction of total-score responsiveness was reproduced, not as an independent replication or equivalence test. Although Group 4 completed the same required course as Group 1, it was not activity-identical because it also included an AI virtual-learner component.

The study was not designed as a randomized efficacy trial. Participants remained in their existing courses, the educational activities differed in content and level across contexts, and no untreated control group was included. Accordingly, pre-test–post-test differences were interpreted as changes occurring during participation in AI-supported educational activities rather than as definitive causal effects of a single standardized intervention. Similarly, differences in the magnitude of pre-test–post-test change across the intact cohorts were interpreted descriptively and were not treated as causal treatment effects.

### 3.2. Participants and Educational Contexts

The analytic sample consisted of 201 participants drawn from four intact educational cohorts that differed in educational level, course purpose, and the structure of their AI-supported learning activities. Groups 1–3 participated during the Fall 2025 semester and represented three concurrent educational contexts: a required-course undergraduate cohort, an elective-course undergraduate cohort, and an in-service teacher cohort in an AI-integrated graduate program. Group 4 participated during the Spring 2026 semester and was treated as a subsequent-semester same-course cohort because it completed the same required course as Group 1 in the following semester; its results were used only to examine directional reproduction of total-score responsiveness.

Across the four cohorts, participants engaged in course-embedded activities involving GenAI-supported learning, instructional design, output evaluation, prompt formulation, revision, and reflection. The instructional emphasis differed by cohort. Groups 1 and 4 focused primarily on AI-supported instructional design in a required undergraduate course. Group 2 used GenAI and related AI tools to develop learning materials in an elective undergraduate course. Group 3 consisted of in-service teachers who designed and evaluated AI-integrated instructional materials for their own educational contexts. These differences allowed the AIES-G to be examined across heterogeneous but conceptually related AI-supported educational settings.

All 201 participants completed both the pre-test and post-test AIES-G and were retained in the matched analyses. The AI-supported activities were conducted as part of regular coursework, whereas participation in the research component was voluntary. All participants provided informed consent before contributing their survey responses to the research (Table 2).

### 3.3. Development of the AI Empathy Scale

#### 3.3.1. Conceptual Specification

The AIES-G was developed following established recommendations for scale construction, including conceptual specification, item generation, content review, pilot testing, and empirical evaluation (Clark & Watson, 2019; Worthington & Whittaker, 2006). The 3 × 2 theoretical structure and six subfactors are developed in detail in Section 2.5; here, the focus is on how those constructs were operationalized for item development. Perspective Taking, Empathic Concern, and Personal Distress retain established multidimensional-empathy terminology (Davis, 1983; Reniers et al., 2011). Imaginative Self-Projection is an author-defined reconstruction of the imaginative/fantasy component for GenAI interaction, whereas Prosocial Intention and Reflective Inquiry are author-defined motivational extensions informed by empathic/prosocial motivation and self-regulated or metacognitive inquiry (Batchelder et al., 2017; Grant, 2008; Panadero, 2017; Weisz & Zaki, 2018).

The scale-development process also drew on the Scientific Empathy Index (Yang et al., 2023), which extended empathy beyond conventional interpersonal targets to students’ disciplinary engagement with scientific phenomena and problems. Rather than transferring that framework through simple target substitution, the present study reconstructed its domain–orientation logic for the interactive, generative, and error-prone characteristics of human–GenAI activity.

#### 3.3.2. Initial Item Pool Generation and Subfactor Operationalization

Items were generated from explicit operational definitions of the six proposed subfactors. PT items assessed the tendency to consider how a GenAI system might interpret a prompt or organize a response. ISP items assessed mental simulation of how one might reason or select information from the position of the AI system. EC items assessed other-oriented concern elicited by limitations, errors, or difficulties arising in AI-mediated interaction. PD items assessed self-focused anxiety, frustration, confusion, or perceived loss of control when a system produced inaccurate or unexpected responses. PI items assessed intentions to improve AI-mediated outcomes, promote responsible use, and support other users through constructive interaction. RI items assessed the motivation to use interaction with GenAI as an opportunity for sustained inquiry, self-explanation, and reconstruction of understanding. Accordingly, the item-generation process combined literature-derived construct functions with study-specific operational definitions adapted to GenAI-mediated interaction; the resulting wording should be understood as a theoretical reconstruction rather than a direct transfer of items from human–human empathy scales.

Based on these operationalizations, an initial candidate pool of 54 items (9 items per subfactor) was generated by the research team. The initial item generation focused specifically on capturing interactive behaviors with GenAI, such as prompt formulation, diagnostic interpretation of output discrepancies, affective responses to hallucinations or system errors, and motivational readiness for iterative refinement.

#### 3.3.3. Content Validity, Expert Review, and Item Refinement

Content validity was evaluated through a two-round Delphi review process with an expert panel consisting of three specialists: one in AI/science education, one in empathy research, and one in educational measurement. The experts evaluated each item on a 4-point Likert scale (1 = not relevant, 4 = highly relevant) in terms of domain relevance, clarity, and theoretical alignment. Because the expert panel consisted of three members, possible I-CVI values were 0.00, 0.33, 0.67, and 1.00. In the present study, agreement by at least two of the three experts (I-CVI ≥ 0.67) was used as the operational criterion for retaining an item for subsequent revision and review, rather than as evidence of definitive content validity. Because the expert panel was small, this operational threshold is more lenient than the I-CVI = 1.00 standard recommended for panels of five or fewer experts (Polit & Beck, 2006); accordingly, the resulting I-CVI values were treated as a screening criterion for item retention rather than as confirmatory evidence of content validity.

Particular attention was given to distinguishing other-oriented affective concern from self-focused distress and to differentiating motivational intention from cognitive understanding or affective response. Through this content evaluation process, 12 items were deleted because of insufficient content validity, unclear wording, or excessive overlap with adjacent constructs such as general AI literacy or metacognitive monitoring. The remaining 42 items were retained, with wording revised where necessary on the basis of expert feedback and subsequent review.

#### 3.3.4. Pilot Testing and Final Instrument Structure

To ensure face validity and comprehensibility, a cognitive interview and pilot test were conducted with a panel of 15 undergraduate and graduate students prior to final administration using a think-aloud protocol. Based on participant feedback, phrasing related to “AI’s perspective” was refined to explicitly denote the “functional interpretation of system prompts and constraints,” thereby preventing anthropomorphic overinterpretation.

This iterative development process resulted in a finalized 42-item instrument containing seven items for each subfactor. One negatively keyed item was included in each subfactor (6 items in total) to control for acquiescence response bias. Participants responded using a 5-point Likert scale ranging from 1 (*strongly disagree*) to 5 (*strongly agree*). Table 3 summarizes the conceptual structure, operational focus, and representative items.

The complete 42-item Korean administered instrument, English translations, and item design focuses are provided in Supplementary Table S4.

### 3.4. Administration and Scoring

Participants completed all 42 items on both measurement occasions using the five-point response scale. The negatively keyed item in each subfactor was reverse-scored using 6 − original response before composite scores were calculated.

Subfactor scores were calculated as the mean of the seven corresponding items. The cognitive, affective, and motivational domain scores were calculated as the means of the 14 items belonging to their two respective subfactors. The total AIES-G score was calculated as the mean of all 42 items. A total score excluding PD (35 items) was also calculated. Using item means retained the original 1–5 response metric and facilitated comparison across subfactors, higher-order domains, and the total scale.

Higher scores on PT, ISP, EC, PI, and RI indicated stronger endorsement of the corresponding AI-empathy disposition. A higher PD score indicated greater self-oriented distress during AI interaction and therefore required substantively different interpretation from the other five subfactors.

### 3.5. Procedure

Across the four cohorts, participants repeatedly interacted with GenAI systems through activities requiring prompt formulation, interpretation of system responses, evaluation of output quality, revision of prompts or outputs, and extension of their initial work. The precise emphasis differed across the educational contexts. Participants were not restricted to a single GenAI platform or underlying model. Consequently, the study reflects ecologically heterogeneous GenAI use rather than responses to one fixed system. Platform- and model-level variation could have contributed to variability in AIES-G scores and should be examined explicitly in future validation studies.

Groups 1–3 completed the study during the Fall 2025 semester. Group 1 focused primarily on AI-assisted lesson design and peer collaboration; Group 2 used GenAI for problem solving within a general-education course; and Group 3 engaged in graduate-level AI-integrated curriculum and instructional design. Group 4 completed the same required course as Group 1 during Spring 2026 and shared the same core AI-assisted lesson-design activities. However, the implementation was not activity-identical: Group 4 additionally included conversations with AI virtual learners. Accordingly, Group 4 should be interpreted as a subsequent-semester same-course cohort with a partially extended activity set, not as an exact procedural replication of Group 1. The common pre-test–post-test administration enabled evaluation of whether the AIES-G detected changes across heterogeneous but conceptually related AI-mediated educational experiences. Because the activities were not standardized as a single intervention, the pooled analysis was designed to assess general scale responsiveness rather than the efficacy of one uniform instructional treatment.

Comparisons among Groups 1–3 examined variation across educational contexts implemented during the same semester. Group 4 was analyzed separately as a subsequent-semester same-course cohort. This distinction was made because educational context and semester could not be estimated as independent effects when Group 4 was the only cohort implemented during Spring 2026; therefore, its results were not treated as evidence of formal replication or equivalence.

### 3.6. Data Preparation

All responses were screened for missing values, invalid response values, duplicate cases, and data-entry errors before analysis. All 201 participants had complete matched pre-test and post-test responses to the 42 AIES-G items; therefore, no item-level imputation was required and no participants were excluded from the primary analyses.

The six negatively keyed items were reverse-scored before subfactor, domain, and total scores were calculated. Descriptive statistics, including means, standard deviations, response-category frequencies, skewness, and kurtosis, were examined for individual items and composite scores. Values were also checked to confirm that all scored item responses remained within the allowable range of 1–5.

### 3.7. Data Analysis

#### 3.7.1. Preliminary Diagnostic Analyses

Because all focal variables were obtained using the same self-report instrument, Harman’s unrotated single-factor procedure was used as a preliminary diagnostic of common method variance. All 42 post-test items were entered into an unrotated factor analysis, and the variance accounted for by the first unrotated factor was examined.

This procedure was treated as a limited diagnostic rather than as definitive evidence that common method bias was absent. A first-factor proportion below the conventional 50% criterion was interpreted only as indicating that the observed item covariance was unlikely to be dominated by a single common factor. Quantitative data were analyzed in jamovi (version 2.3.28). Figures were produced using matplotlib (version 3.10) in Python (version 3.11).

Prior to exploratory factor analysis (EFA), sampling adequacy was evaluated using both the overall Kaiser–Meyer–Olkin (KMO) measure (Kaiser, 1974) and individual item measures of sampling adequacy (MSA). Bartlett’s test of sphericity was conducted to confirm that the correlation matrix was not an identity matrix. The number of factors to retain was determined by integrating three criteria: parallel analysis (PA) with 1000 random datasets using mean eigenvalue thresholds, Velicer’s minimum average partial (MAP) test, and theoretical considerations based on the proposed 3 × 2 framework. EFA was performed using the minimum residual (MINRES) extraction method with oblimin rotation (δ = 0) on Pearson correlations. A sensitivity analysis based on polychoric correlations was conducted to evaluate the robustness of the factor solution to the ordinal response format.

Internal consistency was estimated using both Cronbach’s α and McDonald’s ω for each subscale.

Pre–post changes were evaluated using paired-samples t-tests with 95% confidence intervals and Cohen’s dz as the effect size. For the Personal Distress (PD) subscale, for which little pre-test–post-test change was expected, equivalence was tested using the two one-sided tests (TOST) procedure with ε = ±0.5 SD of the pretest scores. Post hoc power analyses were computed for all pre–post comparisons. A one-way analysis of variance (ANOVA) of difference scores (post minus pre) tested whether pre–post change varied across cohorts (Cohort × Time interaction). Distributional assumptions for the paired-samples t-tests were examined with the Shapiro–Wilk test applied to the difference scores, and Wilcoxon signed-rank tests were conducted as a nonparametric check on every pre–post comparison.

#### 3.7.2. Exploratory Factor Analysis Procedures

Exploratory factor analysis was conducted using the 42 post-test items from all 201 participants to examine the preliminary structural validity of the proposed scale. This corresponds to approximately 4.8 participants per item. In addition, the pooled sample combined four heterogeneous and unequal educational cohorts, with Group 4 contributing 110 of 201 participants (54.7%). Accordingly, the pooled EFA was treated explicitly as preliminary: adequate KMO/MSA values can support factorability but do not eliminate concerns about sample size relative to model complexity or the possibility that cohort heterogeneity contributed to the pooled covariance structure. Post-test data were selected because all participants had completed the relevant GenAI-mediated educational activities by that time and because the post-test represented the intended context of AIES-G use. The EFA procedures, including the selection of the correlation matrix, extraction method, rotation method, and factor-retention criteria, were informed by established recommendations for exploratory factor analysis (Fabrigar et al., 1999; Goretzko et al., 2021). The primary analysis used the Pearson product–moment correlation matrix. Because the five-point items were treated as approximately continuous in this preliminary analysis, the EFA was based on Pearson correlations. The robustness of the solution was examined by repeating the EFA with polychoric correlations, which may be preferable when categorical response distributions depart from approximate continuity (Flora & Curran, 2004). The EFA was also repeated without the six negatively keyed items and with the pre-test items. Factors were extracted using minimum residual estimation and rotated using direct oblimin. Minimum residual extraction was selected because the purpose was to identify common latent factors rather than to summarize total item variance, and because it is less dependent than maximum-likelihood extraction on multivariate-normality assumptions. Oblique rotation was used because the proposed cognitive, affective, and motivational dimensions were theoretically expected to correlate.

The number of factors was evaluated using parallel analysis, Velicer’s minimum average partial criterion (Horn, 1965; Velicer, 1976), examination of the eigenvalue pattern, and theoretical interpretability. The six-factor solution specified by the conceptual 3 × 2 framework was then evaluated in relation to these empirical retention criteria. The EFA was intended to determine whether the theoretically proposed six-factor representation was empirically tenable; it was not treated as confirmatory evidence.

Factorability was evaluated using the Kaiser–Meyer–Olkin measure of sampling adequacy, item-level measures of sampling adequacy, and Bartlett’s test of sphericity. Structural interpretation considered the cumulative proportion of variance explained, the root mean square residual (RMSR), primary pattern loadings, cross-loadings, communalities, factor correlations, and conceptual interpretability.

Absolute pattern loadings of 0.30 or greater were treated as the minimum threshold for a salient item–factor relationship. Items were flagged for closer examination when they showed loadings of 0.30 or greater on multiple factors, when the difference between their two largest absolute loadings was less than 0.20, or when their communalities were comparatively low. Item-retention decisions were interpreted in conjunction with conceptual coverage rather than being based on a single numerical threshold. The complete item-level pattern matrix was retained for reporting in the main manuscript or Supplementary Materials.

#### 3.7.3. Internal Consistency Estimation

Internal consistency was evaluated using Cronbach’s alpha for the total 42-item scale and for each of the six seven-item subfactors at both pre-test and post-test. Reliability was estimated from the same 201 participants on both measurement occasions. McDonald’s omega was also computed for the total scale on both occasions and for the subfactors at post-test.

Estimating reliability at both time points allowed examination of whether item coherence was maintained before and after the AI-supported educational activities. Reliability estimates were interpreted as evidence of score consistency and were considered jointly with the EFA results; they were not treated as evidence of dimensionality by themselves.

#### 3.7.4. Pre-Test–Post-Test Responsiveness

Paired-sample t tests were conducted using the complete sample of 201 participants to examine pre-test–post-test differences in: the total AIES-G score, the three higher-order domain scores, and the six subfactor scores.

Cohen’s dz was calculated by dividing the mean within-person difference by the standard deviation of the difference scores. This effect-size index was selected because the same participants completed both measurements (Lakens, 2013).

Interpretation emphasized the direction and magnitude of change together with exact *p* values. Because multiple related outcomes were examined, the pattern of effects across the theoretically organized domains and subfactors was emphasized rather than interpreting each significance test in isolation.

The pooled analyses were used to evaluate whether the AIES-G detected change across heterogeneous AI-supported educational experiences.

#### 3.7.5. Cohort-Specific and Subsequent-Semester Analyses

Cohort-specific paired-sample t tests were conducted to examine pre-test–post-test change in the total AIES-G score for each of the three Fall 2025 cohorts. The analyses were used to describe whether the direction of total-score responsiveness observed in the pooled sample was also observed across concurrent educational contexts. Group 4 was examined separately because it completed the same mandatory course as Group 1 in the following semester. These within-cohort tests were used only to assess directional reproduction of total-score change; they were not interpreted as demonstrating replication, similarity of effect sizes, or equivalence across cohorts.

#### 3.7.6. Statistical Criteria

All statistical tests were two-tailed, with α set at 0.05. Effect sizes and confidence intervals were considered together with statistical significance. The analyses in this scale-development study were restricted to preliminary structural evidence, internal consistency, pooled pre-test–post-test responsiveness, and descriptive examination of whether total-score responsiveness was directionally reproduced across cohorts and semesters. More detailed analyses of the mechanisms underlying PD change and its associations with the other AI-empathy dimensions were reserved for separate investigation.

## 4. Results

### 4.1. Preliminary Psychometric Evaluation of the AIES-G

Harman’s unrotated single-factor procedure was used as a preliminary diagnostic of common method variance in the 42 post-test items. The first unrotated factor had an eigenvalue of 10.53 and accounted for 25.1% of the total item variance. Thus, no single factor accounted for the majority of item variance. However, Harman’s procedure is an insensitive diagnostic and cannot establish the absence of common method bias; the result was therefore interpreted only as indicating that the observed covariance was not dominated by one unrotated factor (Podsakoff et al., 2003).

### 4.2. Exploratory Factor Analysis

The factorability of the 42 post-test items was examined before interpreting the prespecified six-factor solution. The Kaiser–Meyer–Olkin measure of sampling adequacy was 0.859, and Bartlett’s test of sphericity was statistically significant, χ^2^(861) = 4225.91, *p* < 0.001. These results indicated that the item correlation matrix contained sufficient shared variance for exploratory factor analysis. All 42 items exhibited individual MSA values exceeding the acceptable threshold of 0.70 (range: 0.724–0.931; M = 0.851), confirming that every item contributed adequately to the correlation matrix (Table S2). These diagnostics support the suitability of the pooled correlation matrix for exploratory analysis, but they should not be interpreted as resolving the limited participant-to-item ratio or the unequal contribution of the four cohorts.

Factors were extracted using minimum residual estimation and rotated using direct oblimin rotation. Both parallel analysis and Velicer’s minimum average partial criterion were consistent with the retention of six factors. The six-factor solution accounted for 48.2% of the cumulative item variance, and the root mean square residual (RMSR) for the six-factor MINRES solution was 0.041. However, the number of retained factors did not by itself indicate correspondence with the six intended subfactors.

All 42 items had maximum absolute pattern loadings of at least 0.30, with maximum absolute loadings ranging from 0.327 to 0.793. However, a maximum loading of at least 0.30 does not necessarily indicate that an item loaded most strongly on its theoretically intended factor. Fourteen items were therefore flagged for potential cross-loading or limited factor differentiation: eight had absolute loadings of at least 0.30 on two factors, and six additional items had a difference of less than 0.20 between their two largest absolute loadings. These results indicate limited differentiation among several items.

Correspondence between the intended subfactors and the extracted factors is summarized in Supplementary Table S5. All seven Imaginative Self-Projection items and all seven Personal Distress items loaded primarily on separate factors, as did five of the seven Empathic Concern items. Perspective Taking and Reflective Inquiry items loaded primarily on the same factor. Prosocial Intention did not form a factor of its own, and its items were distributed across four factors. One retained factor consisted mainly of negatively keyed items (PT7, EC7, PI7, and RI7). Another comprised three items from different subfactors (EC6, PI2, and RI3). Thus, two of the six retained factors did not represent intended content dimensions.

When the six negatively keyed items were excluded, both parallel analysis and the MAP criterion indicated five factors. In the five-factor solution for the remaining 36 items (48.6% of cumulative variance; RMSR = 0.044), Imaginative Self-Projection, Empathic Concern, and Personal Distress items again loaded primarily on separate factors. However, three Perspective Taking items loaded with Imaginative Self-Projection, and two Prosocial Intention items loaded with Empathic Concern. The fifth factor again comprised EC6, PI2, and RI3. Perspective Taking, Prosocial Intention, and Reflective Inquiry were not differentiated into distinct factors (Supplementary Table S6). The present items therefore do not empirically support the proposed six-subfactor distinction, particularly within the motivational domain.

Because direct oblimin rotation permits correlated factors, the factor correlation matrix was also examined. Interfactor correlations ranged from −0.068 to 0.422. The strongest correlation was observed between Factor 2 and Factor 3, r = 0.422, followed by Factor 1 and Factor 2, r = 0.302; all remaining absolute correlations were 0.267 or lower. The complete pattern matrix, including all factor loadings, communalities, and cross-loadings, and the complete factor correlation matrix are presented in Supplementary Table S1. Correlations among the six observed subscale scores were examined separately from the interfactor correlations of the rotated solution. These ranged from 0.08 to 0.64 and are displayed in Figure 2.

Item-level measures of sampling adequacy are reported in Supplementary Table S2; all items exceeded the 0.70 threshold, supporting the adequacy of individual items for factor analysis. A sensitivity analysis based on polychoric correlations yielded Tucker’s congruence coefficients of 0.996 to 0.999 with the Pearson-based solution (M = 0.997; Supplementary Table S3), all exceeding the 0.95 threshold (Lorenzo-Seva & ten Berge, 2006). In the pre-test data, parallel analysis and the MAP criterion also indicated six factors. Congruence between the pre-test and post-test solutions was 0.970 for the Empathic Concern factor and 0.981 for the Personal Distress factor, but ranged from 0.752 to 0.859 for the other four factors (Supplementary Table S7). The structure of the cognitive and motivational items may therefore have differed between measurement occasions (Table 4).

### 4.3. Internal Consistency

Internal consistency was high for the total scale and acceptable to good for all six subfactors on both measurement occasions. Cronbach’s alpha for the 42-item AIES-G was 0.923 at pre-test and 0.916 at post-test. At pre-test, subfactor coefficients ranged from 0.762 for Perspective Taking to 0.844 for Imaginative Self-Projection. At post-test, coefficients ranged from 0.756 for Empathic Concern to 0.867 for Imaginative Self-Projection. The broadly comparable coefficients across administrations indicated that the AIES-G scores retained similar levels of internal consistency before and after the AI-supported educational activities. Reliability estimates for the total scale and six subscales are reported in Table 5.

At post-test, McDonald’s omega (ω), which does not assume tau-equivalence, was computed alongside Cronbach’s alpha (α). The two indices showed close agreement across all subscales: PT (α = 0.762, ω = 0.786), ISP (α = 0.867, ω = 0.869), EC (α = 0.756, ω = 0.792), PD (α = 0.831, ω = 0.832), PI (α = 0.776, ω = 0.781), and RI (α = 0.791, ω = 0.802). The close correspondence suggests approximately equal factor loadings within each subscale. For the total scale, ω was 0.949 at pre-test and 0.945 at post-test (Table 6).

### 4.4. Overall, Domain-Level, and Subfactor Changes

Change differed by domain: cognitive empathy showed the largest increase, dz = 1.49, followed by motivational empathy, dz = 1.25, whereas affective empathy increased more modestly, dz = 0.42. At the subfactor level, PT showed the largest change, followed by RI, ISP, PI, and EC. PD was the only subfactor for which no mean change was detected, t(200) = −0.26, *p* = 0.795, dz = −0.02 (Figure 3). Because a nonsignificant paired test does not establish stability, a TOST equivalence test was conducted and showed that the PD difference was statistically equivalent to zero within ε = ±0.365 (0.5 × SD_pre_; t_upper_(200) = −10.56, *p* < 0.001; t_lower_(200) = 10.03, *p* < 0.001), with the 90% CI [−0.068, 0.049] entirely within the equivalence bounds. Post hoc power exceeded 0.99 for all significant comparisons. Shapiro–Wilk tests indicated that the difference scores departed from normality for every subscale (all *p* < 0.001). Wilcoxon signed-rank tests reproduced the pattern obtained with the paired-samples t-tests: all five changing subscales remained significant (all *p* < 0.001), and Personal Distress remained non-significant (*p* = 0.756).

A one-way ANOVA of difference scores tested Cohort × Time interaction. A significant interaction was observed for PI, F(3, 197) = 5.88, *p* < 0.001, indicating that the improvement in Prosocial Intention varied across cohort groups. No significant interactions were observed for the remaining five subscales. Complete descriptive and inferential statistics are reported in Table 7. The pooled results indicated that the AIES-G was responsive across heterogeneous AI-supported educational experiences. The total score excluding PD increased from 2.946 to 3.528, t(200) = 20.67, *p* < 0.001, dz = 1.46 (Table 7).

### 4.5. Cohort-Specific Responsiveness Across Contexts and Semesters

Comparisons across cohorts were used to examine whether the direction of total AIES-G responsiveness was reproduced across educational contexts and semesters. Groups 1–3 were analyzed as concurrent Fall 2025 cohorts because they participated during the same semester but represented different educational contexts. Group 4 was examined as a subsequent-semester same-course cohort because it completed the same required course as Group 1 during Spring 2026. These analyses were descriptive with respect to cross-cohort reproduction and were not treated as tests of replication or equivalence.

All four cohorts showed statistically significant increases in total AIES-G scores (Table 8). Among the Fall 2025 cohorts, Group 1 increased from 3.183 to 3.596, ΔM = 0.412, t(52) = 11.23, *p* < 0.001, dz = 1.54; Group 2 increased from 2.718 to 3.342, ΔM = 0.624, t(19) = 6.09, *p* < 0.001, dz = 1.36; and Group 3 increased from 3.311 to 3.681, ΔM = 0.370, t(17) = 4.62, *p* < 0.001, dz = 1.09. Thus, the AIES-G detected large within-group changes across three concurrent but heterogeneous educational contexts. Ninety-five percent confidence intervals for the mean change were [0.339, 0.486] for Group 1, [0.409, 0.838] for Group 2, [0.201, 0.540] for Group 3, and [0.443, 0.578] for Group 4.

The direction of total-score responsiveness was also observed in the Spring 2026 cohort. Group 4 increased from 2.918 to 3.429, ΔM = 0.511, t(109) = 15.07, *p* < 0.001, dz = 1.44. Because Group 4 participated in a different semester, this finding indicates directional reproduction of within-cohort change only; it does not establish replication, similarity of effect sizes, or equivalence with the Fall 2025 cohorts.

## 5. Discussion

### 5.1. Overview of Findings

Overall, the findings provide preliminary but qualified support for treating AI empathy as a multidimensional human disposition in GenAI-mediated interaction. The number of retained factors matched the conceptual 3 × 2 framework, but the item-level results did not support the six intended subfactors. The present item set therefore requires further refinement. At the same time, the total scale and subscales showed acceptable-to-strong internal consistency. Accordingly, the present findings should be interpreted as preliminary evidence from one exploratory sample rather than as confirmation of the final latent structure.

The responsiveness results were also differentiated rather than uniform: Perspective Taking, Imaginative Self-Projection, Prosocial Intention, and Reflective Inquiry increased more than Empathic Concern, while Personal Distress showed no detected pre-test–post-test change. These findings are interpreted here as evidence of score responsiveness within the observed educational contexts, not as proof of causal instructional effects or as confirmation of a finalized factor structure. The following sections therefore focus on construct boundaries, differentiated responsiveness, and priorities for further validation rather than restating the descriptive results.

### 5.2. Structural Evidence and Construct Boundaries

The number of retained factors matched the conceptual 3 × 2 framework, but the item-level results did not support the six intended subfactors (Section 4.2). The 3 × 2 model is therefore presented as a provisional framework rather than a supported structure. In addition, a maximum loading of 0.30 or higher does not establish that each item loaded most strongly on its theoretically intended factor. Several items showed potential cross-loading or limited factor differentiation, indicating that the present version of the AIES-G should be treated as a preliminary research instrument rather than as a finalized diagnostic scale. The complete pattern matrix and factor correlation matrix therefore remain important for evaluating the extent to which the six conceptual dimensions are empirically differentiated.

The results also suggest that the AIES-G should not be interpreted as either strictly unidimensional or as a set of entirely independent traits. The first unrotated factor accounted for 25.1% of total item variance, indicating meaningful shared variance among the dimensions but not a single dominant factor. A plausible working hypothesis is that AI empathy may involve a broad human-centered disposition that coexists with more specific cognitive, affective, and motivational subfactors. This interpretation remains provisional. Independent studies should compare correlated six-factor, three-domain, higher-order, bifactor, and exploratory structural equation models before stronger claims are made about the hierarchical structure of the scale.

These findings should also be interpreted within the study’s functional and human-centered definition of AI empathy. The AIES-G does not assess empathy possessed by AI, perceived chatbot empathy, or empathy toward AI as a sentient being. Rather, it assesses how human users orient themselves toward AI-mediated tasks, system limitations, their own responses, and the human consequences of AI-generated outputs. This distinction is especially important when interpreting PT and ISP scores: high scores may reflect useful modeling of system behavior, but they may also reflect anthropomorphic overinterpretation if not accompanied by AI literacy, epistemic caution, and critical verification. Therefore, AIES-G profiles should be interpreted as indicators of self-reported orientation and readiness, not as direct evidence of accurate AI understanding or effective AI-use behavior.

### 5.3. Differentiated Cognitive, Affective, and Motivational Responsiveness

The AIES-G showed a large overall pre-test–post-test score increase, but the differentiated pattern across domains is more informative than the total-score change alone. Cognitive scores increased most strongly, motivational scores also showed a large increase, and affective scores changed more modestly. These findings are interpreted as score responsiveness within the observed educational contexts rather than as direct evidence that stable underlying dispositions changed. Here, ‘disposition’ should therefore be understood as a relatively enduring but context-sensitive tendency whose temporal stability remains to be established, not as a fixed trait that is assumed to remain unchanged across learning experiences.

The especially large increase in Perspective Taking scores is consistent with the kinds of activities emphasized in the participating courses, including prompt formulation, interpretation of outputs, and revision. However, this alignment creates an important interpretive ambiguity: the increase may reflect a change in self-reported disposition, but it may also reflect greater familiarity with the task demands, terminology, and response strategies practiced during instruction. The same concern applies, although to different degrees, to Prosocial Intention and Reflective Inquiry because output evaluation, revision, and reflection were themselves part of the learning activities. Accordingly, the present design cannot fully distinguish dispositional change from these instructional confounds, a limitation discussed further, together with additional threats to internal validity, in Section 5.6.

The increases in Prosocial Intention and Reflective Inquiry indicate that AIES-G scores were responsive to the AI-mediated educational experiences, but they should not be interpreted as proof that the courses produced durable motivational-disposition change. PI may overlap with prosocial technology use, and RI may overlap with self-regulated learning, reflective practice, or epistemic agency. Future research should therefore test whether PI and RI demonstrate discriminant and incremental validity beyond these neighboring constructs; predict observable behaviors such as revision, verification, persistence after error, audience-sensitive explanation, and reflective reconstruction; and remain stable over intervals without targeted AI instruction.

The direction of total-score responsiveness was observed across all four cohorts, but this should not be interpreted as formal replication. Groups 1–3 differed in educational level and course purpose, and Group 4 completed the same required course as Group 1 in the following semester while also including an additional AI virtual-learner component. Thus, Group 4 provides a subsequent-semester same-course comparison with partial activity overlap rather than an activity-identical replication. Because the cohorts were intact and nonrandomized, these findings are best interpreted as descriptive evidence of score responsiveness across related educational contexts, not as evidence of equivalent effects or instructional efficacy.

### 5.4. Personal Distress and Interpretation of AIES-G Profiles

Personal Distress requires separate interpretation from the other five subfactors. Whereas higher scores on PT, ISP, EC, PI, and RI are more straightforwardly aligned with constructive engagement, a higher PD score indicates greater self-oriented discomfort, anxiety, frustration, confusion, or perceived loss of control during problematic AI interaction. Therefore, an increase in PD should not be interpreted as a desirable outcome. Although PD is related to emotional discomfort that may subsequently require regulation, it should not be equated with emotion regulation itself, which concerns processes through which individuals influence the occurrence, experience, and expression of emotion (Gross, 2015).

The PD result in the present study should not be interpreted as ‘stability’ on the basis of the nonsignificant paired test alone. Rather, the absence of detected mean change was supplemented by the TOST analysis, which indicated equivalence within the prespecified ±0.5 SD bounds. Within those bounds, participants became more cognitively and motivationally engaged with GenAI without a corresponding increase or decrease in self-oriented distress. This pattern is consistent with the distinction between other-oriented concern and self-focused distress in multidimensional empathy theory and reinforces the importance of interpreting AIES-G profiles at the subfactor level.

The absence of meaningful PD change within the prespecified equivalence bounds also cautions against interpreting AI empathy as a uniformly positive continuum. Unlike PT, ISP, EC, PI, and RI, greater self-oriented distress is not inherently adaptive. This asymmetry suggests that AI empathy may be better conceptualized as a multidimensional dispositional profile rather than as a single desirable trait.

Future research should examine whether PD functions as a boundary condition within AI empathy. For some users, distress may prompt verification, help-seeking, or more careful reflection; for others, it may lead to avoidance, disengagement, or repeated ineffective prompting. The present self-report data cannot distinguish these pathways. The existing GenAI dialogue-log data from participating cohorts are therefore especially important for examining whether PD is associated with observable sequences of error recognition, re-prompting, verification, revision, help-seeking, persistence, or withdrawal.

### 5.5. Implications for Human–AI Interaction and Education

The implications of the present findings extend beyond educational measurement. At a broader behavioral level, the AIES-G provides a framework for examining how individuals differ in interpreting AI behavior, responding to AI-mediated uncertainty, and regulating subsequent interaction. In educational contexts, these differences may be particularly consequential because learners and educators repeatedly decide whether to trust, verify, revise, challenge, or extend AI-generated information.

Recent behavioral evidence further suggests that effective human–AI interaction cannot be inferred from task performance alone. AI-assisted reasoning may improve performance while leaving users less accurate in monitoring the quality of their own judgments, highlighting the importance of self-monitoring and calibrated evaluation during AI use (Fernandes et al., 2026). From this perspective, AIES-G dimensions such as Imaginative Self-Projection, Personal Distress, and Reflective Inquiry may generate testable hypotheses about individual differences in how users recognize uncertainty, reconsider judgments, and verify AI-generated information. These possibilities remain hypotheses, however, until AIES-G scores are linked to observed interaction behavior.

### 5.6. Limitations and Future Validation

Several limitations define the boundaries of the present evidence. First, the structural evidence remains preliminary because the same sample was used to examine factor structure, internal consistency, and responsiveness. The EFA involved 201 participants for 42 items (approximately 4.8 participants per item), and the pooled sample was substantially unbalanced across cohorts, with Group 4 accounting for 54.7% of participants. Because the cohorts differed in educational level, course purpose, and AI-supported activities, some of the pooled covariance structure may reflect between-cohort contextual differences rather than only the intended latent structure. The favorable overall and item-level KMO statistics reduce concerns about factorability but do not remove these design limitations. Item revision should give priority to Prosocial Intention and the negatively keyed items before the structure is tested in a new sample. Future studies should test the proposed structure in larger independent samples and compare correlated six-factor, three-domain, higher-order, bifactor, and exploratory structural equation models. These studies should also use estimators appropriate for ordinal responses and examine measurement invariance across time, cohorts, educational levels, and cultural contexts.

Second, external, behavioral, and incremental validity evidence remains incomplete. Internal consistency demonstrates score coherence, but it does not establish that the AIES-G is empirically distinct from neighboring constructs or predictive of consequential behavior. Future studies should examine associations with AI literacy, anthropomorphism, AI attachment, ASAQ-based evaluations of artificial social agents, critical thinking in AI use, trust in automation, emotion regulation, prosocial orientation, self-regulated learning, reflective practice, epistemic cognition, and conventional empathy measures. A particularly important next step is behavioral validation. The AIES-G assesses dispositions that users report about GenAI-mediated interaction; it does not directly measure what users actually do during interaction. Future studies should therefore connect AIES-G profiles with process-level behavioral data, including prompt sequences, acceptance or rejection of AI outputs, verification behavior, responses to detected errors, re-prompting, revision, persistence, help-seeking, and changes in judgment across interaction turns. Such evidence would allow researchers to examine whether self-reported AI-empathy dispositions predict observable trajectories of human–AI interaction.

Third, the exclusive reliance on self-report introduces risks of common method variance, social desirability, response style, and wording-related effects. Harman’s single-factor procedure indicated that no single unrotated factor accounted for most item variance, but this procedure cannot establish the absence of common method bias. The six negatively keyed items also require further scrutiny because reverse wording may reduce acquiescence while introducing comprehension difficulty or method variance. Cognitive interviews and think-aloud protocols should examine how participants interpret references to the information, context, or conditions that a generative AI system reflects, and whether these expressions create unintended anthropomorphic interpretations.

Fourth, the pre-test–post-test design does not support causal conclusions, and the cultural, contextual, and population generalizability of the AIES-G remains unknown. The study included no untreated comparison group, participants were not randomly assigned, and the educational activities differed across intact cohorts. Moreover, several instructional activities—prompt formulation, output evaluation, revision, and reflection—overlapped directly with processes represented in PT, PI, and RI items. Observed score increases may therefore reflect not only changes in self-reported dispositions but also learned vocabulary, task familiarity, response shift, demand characteristics, maturation, repeated testing, or other unmeasured influences. Because no test–retest interval without targeted GenAI instruction was included, the present data cannot establish the temporal stability of the proposed dispositions or separate trait-like variance from context-sensitive state or learning effects. Future work should include test–retest designs without intervention, controlled or quasi-experimental comparisons where feasible, and samples drawn from noneducational as well as educational contexts. The instrument was developed and administered in Korean; cross-cultural adaptation should therefore involve expert review, cognitive interviewing, and formal tests of measurement invariance. The present study was not preregistered; future confirmatory evaluations of the AIES-G should preregister the hypothesized measurement model, primary psychometric criteria, exclusion rules, and planned responsiveness analyses before data collection.

Taken together, the next phase of validation should focus on three priorities: item and structural refinement, external and behavioral validation, and longitudinal and cross-cultural testing. The current findings support the AIES-G as a promising preliminary instrument for examining how learners and educators orient themselves cognitively, affectively, and motivationally during GenAI-mediated educational activity, but they do not complete the validation of the scale.

## 6. Conclusions

This study developed and preliminarily evaluated the AI Empathy Scale (AIES-G) as a multidimensional measure of human dispositions during GenAI-mediated interaction. Rather than attributing empathy to AI systems, the framework locates AI empathy on the human side of the interaction and treats GenAI as the eliciting context rather than the literal target of empathy. The provisional 3 × 2 framework organizes cognitive, affective, and motivational responses by the locus of attention and regulation, yielding Perspective Taking, Imaginative Self-Projection, Empathic Concern, Personal Distress, Prosocial Intention, and Reflective Inquiry.

The present findings should be regarded as preliminary. Factor-retention procedures supported six factors, but the item-level results did not support the six intended subfactors. The scale nevertheless showed acceptable-to-strong internal consistency and differentiated pre-test–post-test responsiveness. Personal Distress showed no detected mean change and was statistically equivalent to zero within the prespecified TOST bounds, rather than being inferred to be stable from a nonsignificant test alone. These results support item revision followed by confirmatory, convergent, discriminant, behavioral, and cross-context validation.

The AIES-G should nevertheless be regarded as a preliminary research instrument. Its present evidence is based primarily on self-report data from educational contexts, and the proposed structure requires confirmation in independent samples. Future research should establish convergent, discriminant, incremental, behavioral, longitudinal, and cross-cultural validity and determine whether AIES-G profiles predict observable patterns of verification, revision, persistence, help-seeking, and judgment during human–GenAI interaction.

More broadly, the AIES-G provides an initial framework for investigating how people understand AI behavior, respond to AI-mediated uncertainty and consequences, and remain motivated to regulate interaction constructively. As GenAI becomes increasingly embedded in human reasoning, communication, learning, and decision making, examining these cognitive, affective, and motivational differences may contribute to a more comprehensive behavioral understanding of human–AI interaction.

## Figures and Tables

**Figure 1:**
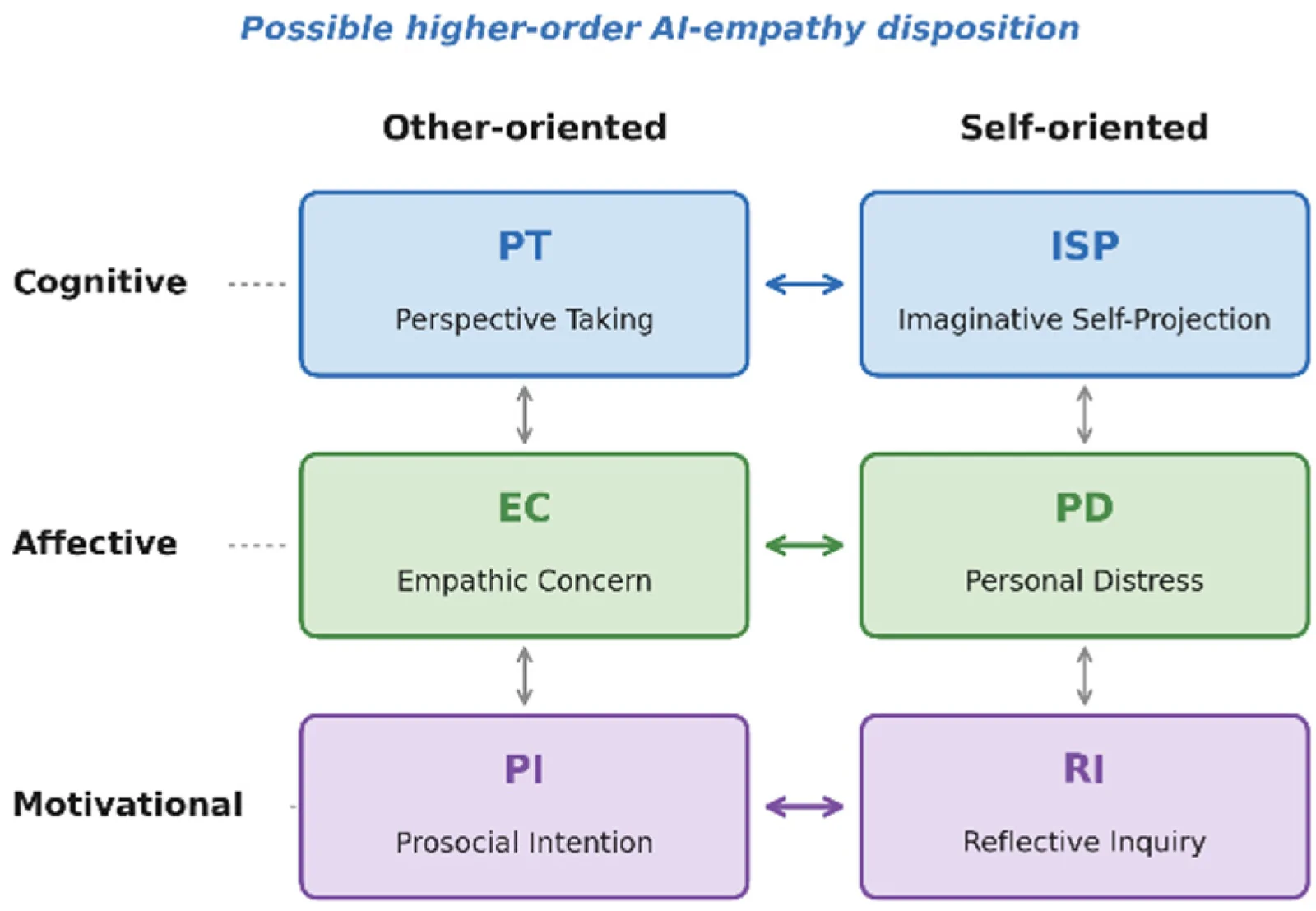
Proposed 3 × 2 model of AI empathy.

**Figure 2:**
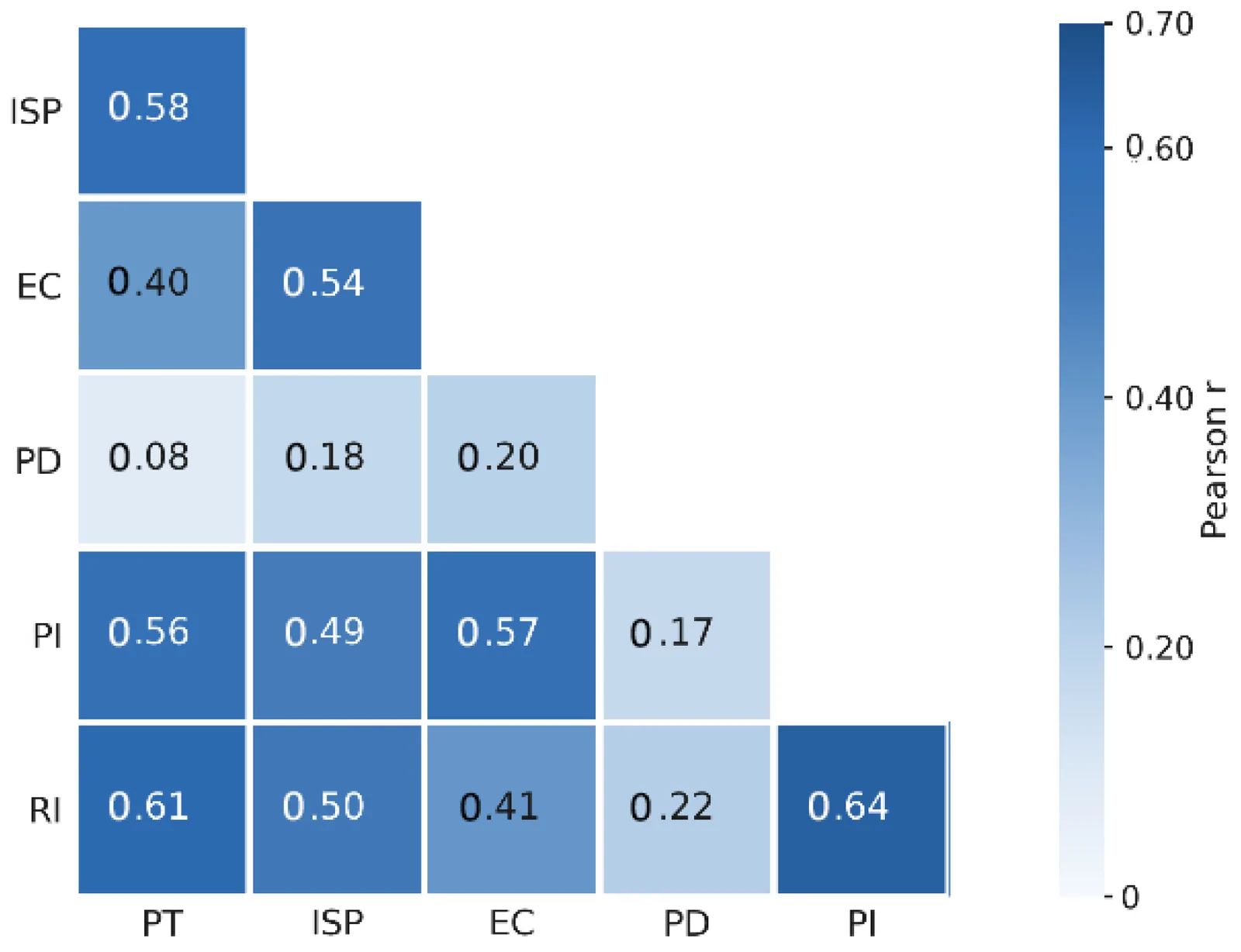
Correlations among the six AIES subfactors at post-test (N = 201). Note. Values are Pearson correlation coefficients. All coefficients are positive and range from 0.08 to 0.64.

**Figure 3:**
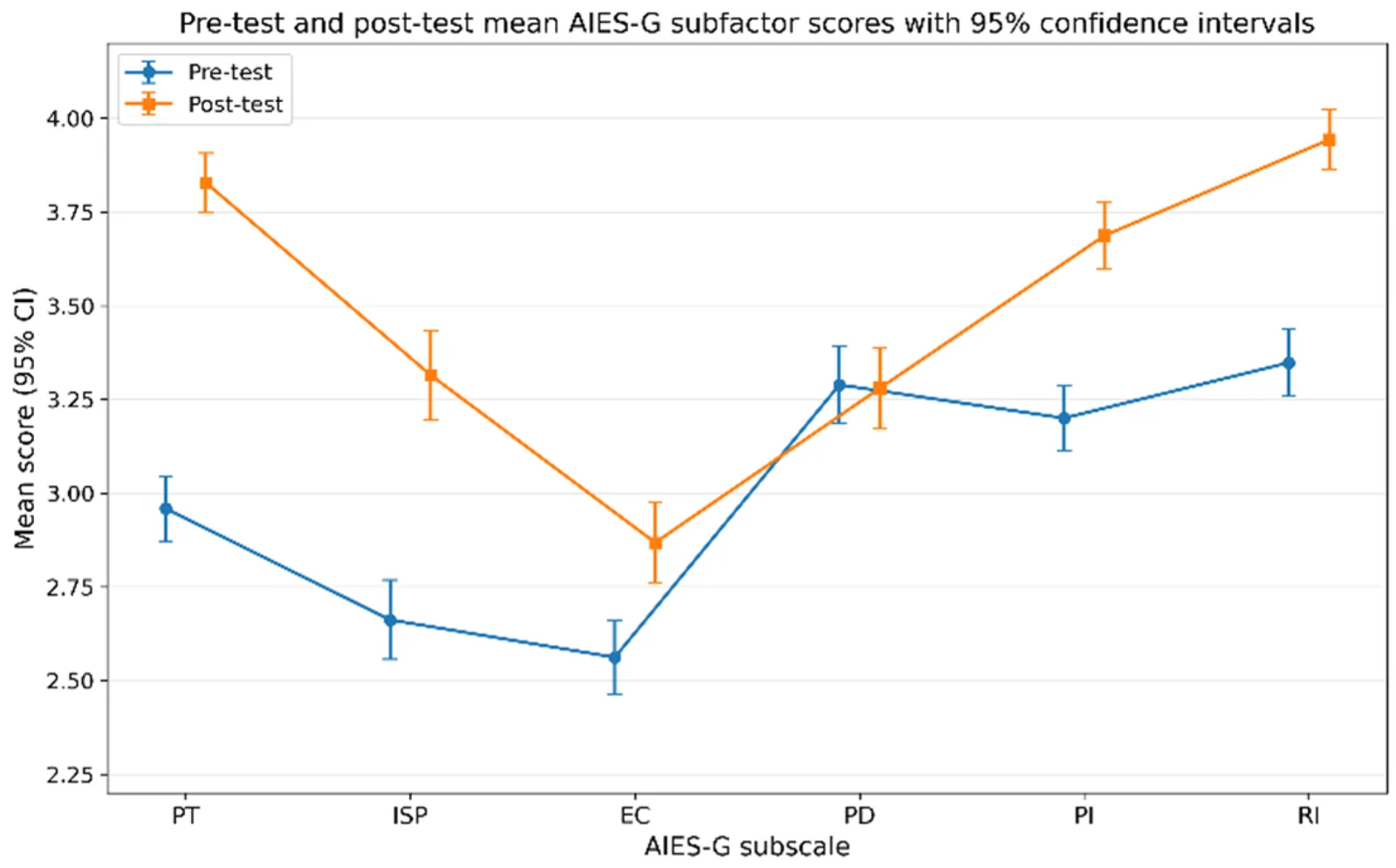
Pre-test and post-test mean AIES-G subfactor scores with 95% confidence intervals. Note. Error bars indicate 95% confidence intervals for the pre-test and post-test mean scores on each AIES-G subfactor (N = 201). Confidence intervals were computed from the observed standard deviations shown in Table 7.

**Table 1:** Conceptual boundaries between AI empathy and adjacent constructs.

Adjacent Construct	Shared Feature	Distinguishing Feature of AI Empathy
AI literacy (Carolus et al., 2023; Long & Magerko, 2020; Ng et al., 2021)	Understanding and evaluating AI capabilities, limitations, and outputs	Includes affective responses and self- and other-oriented motivational readiness in addition to technical and evaluative competence
Anthropomorphism (Epley et al., 2007)	Interpretation of AI as possessing human-like characteristics	Does not require attribution of consciousness, emotion, intention, or a human-like mind
Trust in automation (Hoff & Bashir, 2015)	Evaluation of whether AI outputs should be relied upon	Can involve skepticism, verification, correction, revision, or rejection rather than reliance alone
Self-regulated learning (Panadero, 2017)	Monitoring, persistence, reflection, and strategy adjustment	Includes other-oriented concern and prosocial intention arising specifically in AI-mediated interaction
Emotion regulation (Gross, 2015)	Management of frustration, anxiety, or uncertainty	Represents only part of the affective and regulatory processes within the broader 3 × 2 framework
Prosocial motivation (Grant, 2008)	Intention to support or benefit other people	Is situated specifically in the interpretation, evaluation, and improvement of AI-mediated outcomes
Metacognition (Schraw & Dennison, 1994)	Monitoring and regulation of one’s own thinking	Does not by itself include affective concern, distress, or other-oriented motivational readiness
Epistemic cognition (Greene et al., 2016)	Evaluation of knowledge, evidence, certainty, and justification	Focuses on epistemic judgment, whereas AI empathy additionally includes affective and motivational orientation
Generative AI engagement (Zhang et al., 2025)	Frequency of GenAI use and interaction styles such as Questioningness, Expressiveness, and Preciseness	Focuses on how often and how users interact with GenAI rather than on cognitive, affective, and self-/other-oriented motivational dispositions
AI attachment (Kasturiratna & Hartanto, 2026)	Emotional closeness, social substitution, and normative regard toward AI	Does not require attachment or moral regard toward AI
Critical thinking in AI use (Lau et al., 2026)	Verification, understanding, and reflection in AI use	Adds affective and self- and other-oriented motivational responses
Artificial Social Agent Questionnaire (ASAQ) (Fitrianie et al., 2025)	Evaluation of user experience with artificial social agents across agent and interaction attributes	Focuses on the user’s cognitive, affective, and motivational disposition during GenAI-mediated interaction rather than profiling the social agent

**Table 2:** Participant and cohort characteristics.

Group	N	Semester	Educational Context	Analytic Role
Group 1	53	Fall 2025	Required-course undergraduates	Concurrent-context comparison
Group 2	20	Fall 2025	Elective-course undergraduates	Concurrent-context comparison
Group 3	18	Fall 2025	In-service teachers in a graduate program	Concurrent-context comparison
Group 4	110	Spring 2026	Required-course undergraduates; same core course as Group 1	Subsequent-semester same-course comparison (not activity-identical)

**Table 3:** Conceptual structure and representative items of the AI Empathy Scale for Generative AI Interaction (AIES-G).

Subfactor	Operational Focus	Representative Item
Perspective Taking	A cognitive disposition to reconstruct how prompt information, context, and constraints may have shaped a generative AI response.	I tend to interpret generative AI responses in relation to the prompt and conversation context.
Imaginative Self-Projection	A cognitive disposition to mentally simulate the information, conditions, and reasoning required to construct an appropriate response.	When reading an AI-generated response, I tend to reconstruct for myself what information and conditions would be needed to produce that response.
Empathic Concern	An affective disposition to experience concern about the potential consequences of AI-generated outputs for other people.	I tend to feel concerned when repeated inaccurate AI responses could create difficulties for other users.
Personal Distress	An affective disposition to experience anxiety, discomfort, confusion, or tension in response to erroneous, inconsistent, or unexpected AI-generated outputs.	I tend to feel anxious when generative AI provides an incorrect response.
Prosocial Intention	A motivational disposition to take constructive action so that AI-mediated information or outcomes can be used by other people more accurately, safely, and beneficially.	If the use of an AI-generated response could disadvantage a person or group, I am willing to take steps to reduce that potential harm.
Reflective Inquiry	A motivational disposition to use AI-generated responses, discrepancies, errors, or limitations as prompts for re-examining one’s own questions and judgments and for pursuing further inquiry.	When an AI-generated response differs from my own thinking, I am motivated to re-examine my reasoning and investigate the issue further.

Note. The final instrument contains seven items per subfactor and 42 items in total. One item in each subfactor is negatively keyed. PD represents the intensity of self-oriented distress and should not be interpreted as a uniformly desirable dimension of development.

**Table 4:** Summary of the exploratory factor analysis of post-test AIES-G items (N = 201).

Index	Value
KMO measure of sampling adequacy	0.859
Bartlett’s test of sphericity	χ^2^(861) = 4225.91, *p* < 0.001
Extraction/rotation	Minimum residual/direct oblimin
Parallel analysis	6 factors
Velicer’s MAP criterion	6 factors
Number of factors retained	6
Cumulative variance explained	48.2%
RMSR	0.041
Items with maximum absolute loading ≥ 0.30	42/42
Maximum absolute loading range	0.327–0.793

Note. KMO = Kaiser–Meyer–Olkin; MAP = minimum average partial; RMSR = root mean square residual. Factors were extracted using minimum residual estimation and rotated using direct oblimin rotation. The solution should be regarded as preliminary pending confirmatory evaluation in an independent sample.

**Table 5:** Internal consistency reliability of the AIES-G total scale and subfactors.

Scale	Pre-Test α	Post-Test α
Perspective Taking	0.762	0.762
Imaginative Self-Projection	0.844	0.867
Empathic Concern	0.766	0.756
Personal Distress	0.830	0.831
Prosocial Intention	0.772	0.776
Reflective Inquiry	0.825	0.791
Total	0.923	0.916

**Table 6:** Internal consistency at post-test: Cronbach’s α and McDonald’s ω.

Subscale	Cronbach’s α	McDonald’s ω	Δ (ω − α)
PT	0.762	0.786	0.024
ISP	0.867	0.869	0.002
EC	0.756	0.792	0.035
PD	0.831	0.832	0.001
PI	0.776	0.781	0.006
RI	0.791	0.802	0.011
Total	0.916	0.945	0.029

Note. ω = McDonald’s omega (single-factor model). All values exceed the 0.70 threshold.

**Table 7:** Pre-test–post-test comparison of AIES-G subscale scores with effect sizes and power.

Subscale	Pre M	Pre SD	Post M	Post SD	ΔM	95% CI	t(200)	*p*	dz	Power
PT	2.959	0.622	3.828	0.566	+0.869	[0.793, 0.944]	22.52	<0.001	1.589	>0.999
ISP	2.662	0.758	3.315	0.850	+0.653	[0.574, 0.732]	16.21	<0.001	1.143	>0.999
EC	2.562	0.716	2.868	0.778	+0.306	[0.244, 0.367]	9.73	<0.001	0.687	>0.999
PD	3.289	0.731	3.280	0.773	−0.009	[−0.079, 0.060]	−0.26	0.795	−0.018	0.058
PI	3.200	0.629	3.687	0.641	+0.488	[0.426, 0.549]	15.49	<0.001	1.093	>0.999
RI	3.348	0.638	3.943	0.582	+0.595	[0.526, 0.664]	16.92	<0.001	1.194	>0.999
Total	3.003	0.496	3.487	0.494	+0.483	[0.435, 0.532]	19.62	<0.001	1.384	>0.999
Total excluding PD	2.946	0.534	3.528	0.540	+0.582	[0.527, 0.637]	20.67	<0.001	1.458	>0.999

Note. N = 201. CI = confidence interval. dz = Cohen’s dz (standardized mean difference for paired data). Power was computed using the noncentral t distribution. The pre-test–post-test pattern of subfactor means is visualized in Figure 3.

**Table 8:** Pre-test–post-test change in total AIES-G scores by cohort.

Group	N	Semester	Educational Context	Pre M	Post M	ΔM	t	*p*	dz	95% CI for ΔM
1	53	Fall 2025	Required-course undergraduates	3.183	3.596	+0.412	11.23	<0.001	1.54	[0.339, 0.486]
2	20	Fall 2025	Elective-course undergraduates	2.718	3.342	+0.624	6.09	<0.001	1.36	[0.409, 0.838]
3	18	Fall 2025	In-service teachers in a graduate program	3.311	3.681	+0.370	4.62	<0.001	1.09	[0.201, 0.540]
4	110	Spring 2026	Required-course undergraduates	2.918	3.429	+0.511	15.07	<0.001	1.44	[0.443, 0.578]

Note. Scores are item means on the original 1–5 response scale. Groups 1–3 participated during Fall 2025 and were used for concurrent-context comparison. Group 4 participated during Spring 2026 and was treated as a subsequent-semester same-course cohort used to examine directional reproduction of total-score responsiveness. These comparisons do not constitute tests of replication or equivalence. dz = Cohen’s effect size for paired observations. 95% CI denotes the confidence interval for the within-cohort mean change.

## Data Availability

The original contributions presented in this study are included in the article/Supplementary Materials. Further inquiries can be directed to the corresponding author.

## Supplementary Materials

The following supporting information can be downloaded at: https://www.mdpi.com/article/10.3390/bs16101717/s1, Table S1: Complete pattern matrix for the six-factor exploratory factor analysis of the 42 post-test AIES items (N = 201); Table S2: Item-Level Measures of Sampling Adequacy (MSA);. Table S3: Tucker’s Congruence Coefficients: Pearson vs. polychoric EFA; Table S4: Full 42-item AIES-G: Korean administered items, English translations, and design focus; Table S5: Correspondence Between Intended Subfactors and Empirically Extracted Factors; Table S6: Pattern Matrix for the Five-Factor Exploratory Factor Analysis of the 36 Positively Keyed Post-Test Items (N = 201); Table S7: Tucker’s Congruence Coefficients Between the Pre-Test and Post-Test Six-Factor Solutions.
