# Supplementary material for: AI Empathy as a Human Disposition in Generative AI Interaction: Development and Preliminary Psychometric Evaluation of a Cognitive–Affective–Motivational Scale
Source: Behav Sci (Basel). 2026 Sep 22;16(10):1717. doi: 10.3390/bs16101717 (PMC13701625; doi:10.3390/bs16101717)
Supplement: Supplementary file 1 [file behavsci-16-01717-s001.zip › behavsci-4534205-supplementary.pdf]

Supplementary Table S1

Complete pattern matrix for the six-factor exploratory factor analysis of the 42 post-test AIES items (N = 201)

Analysis information	Value
Extraction / rotation	Minimum residual (MINRES) / direct oblimin equivalent (quartimin, $\delta = 0$)
KMO	.859
Bartlett's test	$\chi^2(861) = 4225.91$, $p < .001$
Minimum item maximum |loading|	.327
Items with maximum |loading| ≥ .30	42 / 42

Panel A. Pattern matrix

Intended subfactor	Item	Factor 1	Factor 2	Factor 3	Factor 4	Factor 5	Factor 6	h^2	u^2	Primary factor	Primary loading	Loading gap	Cross-loading flag
Perspective Taking	PT1	**.560**	.123	--.027	.046	.066	--.069	.400	.600	F1	.560	.437	No
Perspective Taking	PT2	**.469**	**.324**	--.004	--.072	--.026	--.012	.393	.607	F1	.469	.145	Yes
Perspective Taking	PT3	**.367**	**.328**	--.010	--.173	.006	.022	.312	.688	F1	.367	.040	Yes
Perspective Taking	PT4	**.432**	**.369**	.132	--.062	--.021	.042	.493	.507	F1	.432	.064	Yes
Perspective Taking	PT5	**.415**	.192	.001	--.052	--.017	--.079	.246	.754	F1	.415	.223	No
Perspective Taking	PT6	**.611**	.176	--.048	--.014	.035	.014	.463	.537	F1	.611	.435	No
Perspective Taking	PT7	.176	.032	--.061	--.109	**.419**	--.063	.237	.763	F5	.419	.243	No
Imaginative Self-Projection	ISP1	.211	**.671**	--.116	.084	--.173	.002	.507	.493	F2	.671	.460	No
Imaginative Self-Projection	ISP2	.273	**.622**	.030	--.020	--.106	.009	.553	.447	F2	.622	.349	No
Imaginative Self-Projection	ISP3	.056	**.502**	.100	.057	.104	.195	.439	.561	F2	.502	.307	No
Imaginative Self-Projection	ISP4	.051	**.717**	.134	.053	.050	.028	.677	.323	F2	.717	.583	No
Imaginative Self-Projection	ISP5	.120	**.658**	.077	.049	.010	--.026	.560	.440	F2	.658	.538	No
Imaginative Self-Projection	ISP6	.268	**.357**	.248	.084	.121	--.171	.507	.493	F2	.357	.090	Yes
Imaginative Self-Projection	ISP7	--.246	**.691**	.033	--.013	**.421**	--.026	.683	.317	F2	.691	.270	Yes
Empathic Concern	EC1	--.083	.112	**.768**	--.037	--.021	.042	.654	.346	F3	.768	.656	No
Empathic Concern	EC2	.198	--.019	**.653**	.151	.043	**--.437**	.674	.326	F3	.653	.215	Yes
Empathic Concern	EC3	.052	--.069	**.793**	--.057	.041	.149	.676	.324	F3	.793	.644	No
Empathic Concern	EC4	--.056	.124	**.760**	--.027	.007	.194	.741	.259	F3	.760	.566	No
Empathic Concern	EC5	.254	--.004	**.396**	.235	.054	**--.316**	.436	.564	F3	.396	.080	Yes
Empathic Concern	EC6	--.046	.047	.229	.043	--.050	**.757**	.687	.313	F6	.757	.528	No
Empathic Concern	EC7	.052	.094	.118	.246	**.351**	--.262	.357	.643	F5	.351	.089	Yes
Personal Distress	PD1	--.028	.028	.045	**.736**	--.015	.009	.541	.459	F4	.736	.691	No
Personal Distress	PD2	.081	--.170	**--.322**	**.600**	.195	--.036	.560	.440	F4	.600	.278	Yes
Personal Distress	PD3	.065	--.043	--.035	**.714**	--.075	--.046	.520	.480	F4	.714	.639	No
Personal Distress	PD4	.135	--.170	--.256	**.640**	.084	--.005	.539	.461	F4	.640	.384	No
Personal Distress	PD5	--.082	.170	.140	**.700**	--.171	.135	.558	.442	F4	.700	.529	No
Personal Distress	PD6	--.082	.234	.135	**.618**	--.042	.099	.488	.512	F4	.618	.384	No
Personal Distress	PD7	--.141	.091	.037	**.541**	.225	--.062	.387	.613	F4	.541	.316	No
Prosocial Intention	PI1	.083	.083	**.389**	.008	.201	.299	.429	.571	F3	.389	.089	Yes
Prosocial Intention	PI2	.256	--.122	.154	.103	.152	**.575**	.514	.486	F6	.575	.319	No
Prosocial Intention	PI3	**.327**	.057	.233	.040	.289	.215	.468	.532	F1	.327	.038	Yes
Prosocial Intention	PI4	**.479**	--.014	.048	.017	.198	.128	.360	.640	F1	.479	.280	No
Prosocial Intention	PI5	.285	.090	**.391**	--.025	.039	.218	.434	.566	F3	.391	.106	Yes
Prosocial Intention	PI6	**.447**	--.047	.007	.075	.284	.034	.362	.638	F1	.447	.163	Yes
Prosocial Intention	PI7	.146	--.003	.025	--.071	**.604**	.071	.436	.564	F5	.604	.458	No
Reflective Inquiry	RI1	**.585**	.014	.111	.099	.051	--.076	.444	.556	F1	.585	.474	No
Reflective Inquiry	RI2	**.515**	--.039	--.035	.040	.082	.170	.322	.678	F1	.515	.345	No
Reflective Inquiry	RI3	**.386**	.130	--.076	.052	.059	**.462**	.441	.559	F6	.462	.076	Yes
Reflective Inquiry	RI4	**.562**	--.008	--.064	--.003	.020	.290	.404	.596	F1	.562	.272	No
Reflective Inquiry	RI5	**.569**	.050	.098	.059	.122	.002	.460	.540	F1	.569	.447	No
Reflective Inquiry	RI6	**.553**	.110	.151	.030	.068	--.225	.490	.510	F1	.553	.329	No
Reflective Inquiry	RI7	.152	.104	0	.045	**.532**	.008	.404	.596	F5	.532	.381	No

Panel B. Factor correlation matrix (Φ)

Factor	Factor 1	Factor 2	Factor 3	Factor 4	Factor 5	Factor 6
Factor 1	1	.302	.224	.188	.267	.058
Factor 2	.302	1	.422	.085	.186	.095

Factor 3	.224	.422	1	.069	.157	.162
Factor 4	.188	.085	.069	1	.158	--.068
Factor 5	.267	.186	.157	.158	1	.015
Factor 6	.058	.095	.162	--.068	.015	1

Note. Pattern coefficients are reported. Absolute coefficients ≥ .30 are highlighted. h^2 = communality; u^2 = uniqueness. A cross-loading flag is shown when at least two absolute loadings are ≥ .30 or the gap between the two largest absolute loadings is < .20. Factor order and signs were rearranged only for interpretability; this does not change the statistical solution.

Supplementary Table S2

Item-Level Measures of Sampling Adequacy (MSA)

Item	MSA		Item	MSA		Item	MSA
PT1	0.913		EC1	0.841		PI1	0.896
PT2	0.915		EC2	0.877		PI2	0.829
PT3	0.859		EC3	0.866		PI3	0.904
PT4	0.931		EC4	0.862		PI4	0.890
PT5	0.807		EC5	0.841		PI5	0.840
PT6	0.909		EC6	0.757		PI6	0.855
PT7(R)	0.724		EC7(R)	0.832		PI7(R)	0.850
ISP1	0.828		PD1	0.741		RI1	0.887
ISP2	0.924		PD2	0.745		RI2	0.788
ISP3	0.894		PD3	0.757		RI3	0.873
ISP4	0.891		PD4	0.725		RI4	0.837
ISP5	0.920		PD5	0.781		RI5	0.892
ISP6	0.906		PD6	0.823		RI6	0.867
ISP7(R)	0.853		PD7(R)	0.824		RI7(R)	0.828

Note. N = 201. Overall KMO = .859. All items exceed the .70 threshold. Bartlett's test of sphericity: $\chi^2(861) = 4225.91$, $p < .001$.

Supplementary Table S3

Tucker's Congruence Coefficients: Pearson vs. polychoric EFA

Factor	Tucker's φ	Interpretation
F1 (PT/RI)	0.997	Equivalent
F2 (ISP)	0.996	Equivalent
F3 (EC)	0.998	Equivalent
F4 (PD)	0.999	Equivalent
F5 (R-items)	0.997	Equivalent
F6 (Misc.)	0.996	Equivalent
**Mean**	**0.997**	**Equivalent**

Note. φ ≥ .95 indicates factor equivalence (Lorenzo-Seva & ten Berge, 2006). All factors exceed this threshold. Factor numbering follows Table S1.

A sensitivity analysis based on polychoric correlations yielded Tucker's congruence coefficients of .996 to .999 with the Pearson-based solution (M = .997; Table S3), all exceeding the .95 threshold for factor equivalence. The factor structure was not dependent on treating the items as continuous.

Supplementary Table S4

Full 42-item AIES-G: Korean administered items, English translations, and design focus.

Item	Korean administered item	English translation	Design focus
PT1	나는 프롬프트를 작성할 때 생성형 AI 가 어떤 정보와 조건을 반영할지를 고려하는 편이다.	I tend to consider what information and conditions generative AI may take into account when I formulate a prompt.	Prompt/context condition consideration
PT2	나는 생성형 AI 의 응답이 어떤 정보와 맥락에 근거해 생성되었는지 추론해 보려는 편이다.	I tend to infer what information and context may have contributed to a generative AI response.	Response-basis inference
PT3	생성형 AI 의 응답이 내 판단과 다를 때에도, 그 차이가 어떤 정보나 조건에서 비롯되었는지 살펴보려는 편이다.	Even when a generative AI response differs from my judgment, I tend to examine what information or conditions may account for the difference.	Interpretation of response–judgment discrepancy
PT4	생성형 AI 의 응답에 환각이나 오류가 나타나면, 어떤 맥락이나 조건이 관련되었을지 살펴보는 편이다.	When generative AI produces a hallucination or error, I tend to examine what contextual information or conditions may have contributed to it.	Functional interpretation of error conditions
PT5	나는 여러 생성형 AI 의 응답을 비교하면서 각 시스템이 어떤 정보나 조건을 다르게 반영했는지 살펴보는 편이다.	When comparing responses from different generative AI systems, I tend to examine what information or conditions each system reflected differently.	Cross-system contextual comparison
PT6	나는 생성형 AI 의 응답을 프롬프트와 대화 맥락을 함께 고려하여 해석하는 편이다.	I tend to interpret generative AI responses in relation to the prompt and conversation context.	Context-sensitive response interpretation
PT7-R	(R) 나는 생성형 AI 의 응답을 판단할 때, 그 응답이 만들어진 맥락이나 조건은 거의 고려하지 않는 편이다.	(R) When judging a generative AI response, I rarely consider the context or conditions under which the response was generated.	Reverse: neglect of contextual conditions

ISP1	나는 생성형 AI 가 적절한 답변을 만들기 위해 어떤 정보가 필요할지 머릿속으로 구성해 보는 편이다.	I tend to imagine what information a generative AI system would need to produce an appropriate response.	Simulation of required information
ISP2	나는 적절한 응답을 구성하는 데 필요한 정보와 추론을 머릿속으로 시뮬레이션해 보는 편이다.	I tend to mentally simulate the information and reasoning that would be needed to construct an appropriate response.	Self-oriented simulation of response requirements; removes AI-internal-process wording
ISP3	생성형 AI 의 응답을 읽을 때, 그 답변을 만들기 위해 어떤 정보와 조건이 필요했을지 스스로 재구성해 보는 편이다.	When reading an AI-generated response, I tend to reconstruct for myself what information and conditions would be needed to produce that response.	Mental reconstruction of response requirements
ISP4	같은 문제에 대한 응답을 만들어야 한다면 어떤 정보와 판단 기준이 필요할지 상상해 보는 편이다.	When considering how to respond to the same problem, I tend to imagine what information and decision criteria would be needed.	Simulation of task and decision requirements
ISP5	나는 생성형 AI 의 응답 방식을 참고하여 문제를 다른 방식으로 구성해 보는 편이다.	I sometimes use the structure of an AI-generated response to reconstruct the problem in a different way.	Problem reconstruction through response structure
ISP6	문제를 해결할 때 생성형 AI 가 더 적절하게 답변하려면 어떤 정보가 추가로 필요할지 상상해 보는 편이다.	When solving a problem, I tend to imagine what additional information would be needed for generative AI to provide a more appropriate response.	Simulation of missing information requirements
ISP7-R	(R) 나는 생성형 AI 응답에 필요한 정보나 조건을 머릿속으로 시뮬레이션하는 경우가 거의 없다.	(R) I rarely mentally simulate the information or conditions needed to generate an AI response.	Reverse: absence of mental simulation
EC1	생성형 AI 의 오류로 인해 다른 사용자가 잘못된 정보를 얻을 수 있다고 생각하면 걱정되는 편이다.	I tend to feel concerned when an error in a generative AI response could lead another user to incorrect information.	Concern about misinformation affecting others
EC2	생성형 AI 가 반복적으로 부정확한 응답을 제공하여 다른 사용자가 어려움을 겪을 수 있을 때 걱정되는 편이다.	I tend to feel concerned when repeated inaccurate AI responses could create difficulties for other users.	Concern about repeated-error consequences
EC3	생성형 AI 의 부적절한 응답으로 다른 사람이 혼란이나 불이익을 겪는 상황에 마음이 쓰이는 편이다.	I tend to feel concerned when an inappropriate AI-generated response causes confusion or disadvantage for another person.	Concern about another person's confusion/disadvantage
EC4	생성형 AI 의 한계 때문에 도움이 필요한 사람이 적절한 지원을 받지 못할 수 있는 상황을 걱정하는 편이다.	I tend to worry when limitations in generative AI may prevent someone from receiving the support they need.	Concern about unmet human support needs
EC5	생성형 AI 가 사용자의 상황이나 어려움을 충분히 반영하지 못한 응답을 제공할 때 그 사용자에게 미칠 영향을 염려하는 편이다.	When generative AI does not adequately reflect a user's situation or difficulty, I tend to be concerned about the effect on that user.	Concern about insufficient contextual responsiveness
EC6	생성형 AI 의 편향된 응답이 특정 사용자나 집단에 부정적인 영향을 줄 수 있다고 생각하면 걱정되는 편이다.	I tend to feel concerned when biased AI-generated responses could negatively affect particular users or groups.	Concern about bias-related human consequences

EC7-R	(R) 생성형 AI 의 부적절한 응답이 다른 사람에게 부정적인 영향을 줄 수 있어도 나는 별로 걱정하지 않는 편이다.	(R) Even when an inappropriate AI-generated response could negatively affect another person, I tend not to feel much concern.	Reverse: low concern for affected others
PD1	나는 생성형 AI 가 틀린 응답을 제공하면 불안해지는 편이다.	I tend to feel anxious when generative AI provides an incorrect response.	Anxiety in response to AI error
PD2	나는 생성형 AI 의 응답이 비논리적이거나 앞뒤가 맞지 않을 때 답답함을 느끼는 편이다.	I tend to feel frustrated when a generative AI response is illogical or internally inconsistent.	Frustration with illogical/inconsistent output
PD3	생성형 AI 의 오류를 발견하면 마음이 불편해지는 편이다.	I tend to feel uncomfortable when I detect an error in a generative AI response.	Discomfort following error detection
PD4	생성형 AI 가 같은 오류를 반복하면 불편감이 커지는 편이다.	I tend to feel increasingly uncomfortable when generative AI repeatedly produces the same error.	Distress from repeated error
PD5	생성형 AI 의 응답을 이해하기 어려울 때 감정적으로 혼란스러워지는 편이다.	I tend to feel emotionally confused when a generative AI response is difficult to understand.	Emotional confusion under incomprehensibility
PD6	생성형 AI 의 응답이 예상과 크게 다르면 긴장되는 편이다.	I tend to feel tense when a generative AI response differs substantially from what I expected.	Tension from expectation discrepancy
PD7-R	(R) 생성형 AI 가 부정확하거나 비정상적인 응답을 보여도 나는 별로 불편하지 않은 편이다.	(R) I tend not to feel much discomfort even when generative AI produces inaccurate or abnormal responses.	Reverse: low self-directed distress
PI1	생성형 AI 의 응답이 다른 사람의 필요나 상황을 충분히 반영하지 못하면, 그 사람에게 더 도움이 되도록 응답을 개선하기 위해 행동하려는 편이다.	When an AI-generated response does not adequately address another person's needs or circumstances, I am willing to take steps to make the response more useful for that person.	Human beneficiary + action
PI2	나는 다른 사람들이 생성형 AI 의 정보를 정확하고 안전하며 유익하게 활용할 수 있도록 돕고 싶은 편이다.	I am willing to help others use AI-generated information in ways that are accurate, safe, and beneficial.	Generalized prosocial readiness
PI3	다른 사람이 활용할 수 있는 생성형 AI 응답에서 오류나 오해의 소지가 있는 정보를 발견하면, 그들이 잘못된 정보를 활용할 위험을 줄이기 위해 행동하려는 편이다.	When I notice an error or misleading information in an AI-generated response that others might use, I am willing to take action to reduce the risk of their being misled.	Error → other-protective action
PI4	나는 주변 사람들이 생성형 AI 를 보다 적절하고 책임 있게 활용할 수 있도록 돕고 싶은 편이다.	I am willing to help people around me use generative AI more appropriately and responsibly.	Direct helping intention
PI5	생성형 AI 의 정보를 다른 사람과 공유할 때, 그들이 정보를 더 적절하게 활용하는 데 도움이 된다면 그 한계도 함께 설명하려는 편이다.	When sharing AI-generated information with others, I am willing to clarify its limitations when doing so could help them use it more appropriately.	Concrete prosocial communication
PI6	생성형 AI 의 응답을 활용하는 것이 특정 사람이나 집단에 불리한 결과를 초래할 수 있다면, 그러한 잠재적 피해를 줄이기 위해 행동하려는 편이다.	If the use of an AI-generated response could disadvantage a person or group, I am willing to take steps to reduce that potential harm.	Harm-reduction readiness

PI7-R	(R) 생성형 AI 의 응답이 다른 사람에게 문제를 일으킬 수 있어도, 나는 이를 위해 어떤 행동을 해야겠다는 동기를 거의 느끼지 않는 편이다.	(R) Even when an AI-generated response could cause problems for other people, I rarely feel motivated to do anything about it.	Clean reverse of prosocial action
RI1	생성형 AI 의 응답이 내 생각과 다를 때, 나는 내 사고 과정을 다시 검토하고 그 문제를 더 탐구하려는 편이다.	When an AI-generated response differs from my own thinking, I am motivated to re-examine my reasoning and investigate the issue further.	Discrepancy → inquiry
RI2	생성형 AI 의 응답에서 익숙하지 않은 관점을 접하면, 나는 처음 받은 응답에 그치지 않고 그 문제를 더 탐구하려는 편이다.	When an AI-generated response introduces an unfamiliar perspective, I am motivated to investigate the issue beyond the initial response.	New perspective → further inquiry
RI3	생성형 AI 의 응답에서 오류를 발견하면, 나는 내 질문이나 판단을 다시 검토하고 추가 근거를 찾아보려는 편이다.	When I detect an error in an AI-generated response, I am motivated to reconsider my question or judgment and seek additional evidence.	Error → self-check + evidence
RI4	생성형 AI 의 응답이 내가 예상한 것과 다를 때, 나는 기존의 이해를 수정할 필요가 있는지 더 탐구해 보려는 편이다.	When an AI-generated response challenges what I expected, I am motivated to explore why my initial understanding may need revision.	Challenge → revision inquiry
RI5	예상하지 못한 생성형 AI 의 응답을 접하면, 나는 후속 탐구를 위한 새로운 질문을 만들어 보려는 편이다.	An unexpected AI-generated response often motivates me to develop new questions for further inquiry.	Generative inquiry
RI6	생성형 AI 응답의 한계를 발견하면, 나는 내가 더 알아보거나 탐구해야 할 부분을 찾아보려는 편이다.	When I recognize a limitation in an AI-generated response, I am motivated to identify what I still need to learn or investigate.	Limitation → knowledge gap
RI7-R	(R) 생성형 AI 의 응답에서 새로운 의문이나 한계를 발견해도, 나는 추가로 탐구하려는 동기를 거의 느끼지 않는 편이다.	(R) Even when an AI-generated response raises a new question or reveals a limitation, I rarely feel motivated to investigate further.	Clean reverse

Note. The psychometric analyses reported in this study were based on the Korean version administered to participants. The English items are translations provided for reporting purposes. Where necessary, the English wording was refined to improve semantic equivalence with the administered Korean items without altering the content of the administered instrument. One item in each subfactor is reverse-keyed and is marked "R."

Supplementary Table S5

Correspondence Between Intended Subfactors and Empirically Extracted Factors

Intended subfactor	Modal factor	Items on modal factor	Distribution of primary loadings
Perspective Taking	F1	6 / 7	F1: 6, F5: 1
Imaginative Self-Projection	F2	7 / 7	F2: 7
Empathic Concern	F3	5 / 7	F3: 5, F5: 1, F6: 1
Personal Distress	F4	7 / 7	F4: 7
Prosocial Intention	F1	3 / 7	F1: 3, F3: 2, F5: 1, F6: 1
Reflective Inquiry	F1	5 / 7	F1: 5, F5: 1, F6: 1

Note. Primary loadings are taken from the pattern matrix in Table S1. The modal factor is the extracted factor on which the largest number of a subfactor's seven items loaded most strongly. Factor numbering follows Table S1. Four of the six negatively keyed items (PT7, EC7, PI7, and RI7) loaded most strongly on F5.

Supplementary Table S6

Pattern Matrix for the Five-Factor Exploratory Factor Analysis of the 36 Positively Keyed Post-Test Items (N = 201)

Intended subfactor	Item	F1	F2	F3	F4	F5	h²	Primary factor
Perspective Taking	PT1	.528	.202	−.051	.022	−.088	.391	F1
Perspective Taking	PT2	.388	.404	−.034	−.096	−.037	.399	F2
Perspective Taking	PT3	.294	.408	−.043	−.184	.017	.325	F2
Perspective Taking	PT4	.338	.452	.098	−.079	.034	.490	F2
Perspective Taking	PT5	.353	.251	−.025	−.064	−.087	.240	F1
Perspective Taking	PT6	.554	.271	−.069	−.028	−.015	.457	F1
Imaginative Self-Projection	ISP1	.020	.738	−.155	.074	−.005	.492	F2
Imaginative Self-Projection	ISP2	.105	.708	−.010	−.022	.002	.558	F2
Imaginative Self-Projection	ISP3	.050	.511	.115	.064	.187	.420	F2
Imaginative Self-Projection	ISP4	−.013	.720	.147	.064	.011	.643	F2
Imaginative Self-Projection	ISP5	.033	.675	.078	.055	−.037	.536	F2
Imaginative Self-Projection	ISP6	.262	.369	.263	.087	−.191	.497	F2
Empathic Concern	EC1	−.105	.124	.752	−.037	.046	.644	F3
Empathic Concern	EC2	.183	.015	.640	.142	−.460	.671	F3
Empathic Concern	EC3	.093	−.065	.800	−.064	.126	.677	F3
Empathic Concern	EC4	−.055	.129	.758	−.027	.183	.736	F3
Empathic Concern	EC5	.242	.033	.395	.233	−.350	.448	F3
Empathic Concern	EC6	−.023	.049	.230	.042	.754	.688	F5
Personal Distress	PD1	−.039	.039	.044	.750	.001	.564	F4
Personal Distress	PD2	.219	−.210	−.284	.588	−.048	.531	F4
Personal Distress	PD3	.051	−.025	−.056	.674	−.051	.474	F4
Personal Distress	PD4	.220	−.190	−.237	.632	−.021	.547	F4
Personal Distress	PD5	−.187	.220	.109	.700	.121	.562	F4
Personal Distress	PD6	−.110	.230	.144	.624	.072	.493	F4
Prosocial Intention	PI1	.186	.071	.418	.012	.287	.411	F3
Prosocial Intention	PI2	.370	−.116	.183	.103	.547	.512	F5
Prosocial Intention	PI3	.457	.052	.283	.058	.167	.441	F1
Prosocial Intention	PI4	.563	.004	.077	.023	.091	.362	F1
Prosocial Intention	PI5	.297	.114	.389	−.036	.198	.433	F3
Prosocial Intention	PI6	.566	−.026	.047	.085	−.016	.349	F1
Reflective Inquiry	RI1	.568	.083	.096	.076	−.116	.436	F1
Reflective Inquiry	RI2	.550	.002	−.040	.016	.148	.323	F1
Reflective Inquiry	RI3	.412	.167	−.072	.041	.436	.441	F5
Reflective Inquiry	RI4	.540	.073	−.089	−.023	.270	.382	F1
Reflective Inquiry	RI5	.583	.103	.101	.057	−.036	.458	F1
Reflective Inquiry	RI6	.504	.195	.127	.015	−.247	.473	F1

Factor correlation matrix (Φ)

Factor	F1	F2	F3	F4	F5
F1	1	.376	.214	.206	.027
F2	.376	1	.444	.094	.087
F3	.214	.444	1	.061	.154
F4	.206	.094	.061	1	−.063
F5	.027	.087	.154	−.063	1

Note. The six negatively keyed items (PT7, ISP7, EC7, PD7, PI7, and RI7) were excluded. Parallel analysis (mean eigenvalues from 1,000 random data sets) and Velicer's MAP criterion both indicated five factors. Factors were extracted using minimum residual estimation with direct oblimin rotation. The solution accounted for 48.6% of the cumulative item variance (RMSR = .044). F1 comprised mainly Reflective Inquiry, Prosocial Intention, and Perspective Taking items; F2, Imaginative Self-Projection and Perspective Taking items; F3, Empathic Concern and Prosocial Intention items; F4, Personal Distress items; and F5, EC6, PI2, and RI3.

Supplementary Table S7

Tucker's Congruence Coefficients Between the Pre-Test and Post-Test Six-Factor Solutions

Factor	Tucker's φ	Interpretation
F1 (PT/RI)	0.809	Low similarity
F2 (ISP)	0.859	Fair similarity
F3 (EC)	0.970	Equivalent
F4 (PD)	0.981	Equivalent
F5 (R-items)	0.752	Low similarity
F6 (Misc.)	0.770	Low similarity
Mean	0.857	

Note. The same EFA specification (42 items, minimum residual extraction, direct oblimin rotation, six factors) was applied to the pre-test items. Parallel analysis and Velicer's MAP criterion also indicated six factors in the pre-test data. Pre-test factors were matched to the post-test factors in Table S1 by maximizing absolute congruence; absolute values are reported. $\varphi \geq .95$ indicates equivalence, .85 to .94 indicates fair similarity, and values below .85 indicate low similarity (Lorenzo-Seva & ten Berge, 2006).
